# Bacterial Vipp1 and PspA are members of the ancient ESCRT-III membrane-remodeling superfamily

**DOI:** 10.1016/j.cell.2021.05.041

**Published:** 2021-07-08

**Authors:** Jiwei Liu, Matteo Tassinari, Diorge P. Souza, Souvik Naskar, Jeffrey K. Noel, Olga Bohuszewicz, Martin Buck, Tom A. Williams, Buzz Baum, Harry H. Low

**Affiliations:** 1 Department of Infectious Disease, Imperial College, London, UK; 2 MRC Laboratory for Molecular Cell Biology, University College London, London, UK; 3 Division of Cell Biology, MRC Laboratory of Molecular Biology, Cambridge, UK; 4 Institute for the Physics of Living Systems, University College London, London, UK; 5 Max Delbrück Center for Molecular Medicine, Berlin, Germany; 6 Department of Life Sciences, Imperial College, London, UK; 7 School of Biological Sciences, University of Bristol, Bristol, UK

**Keywords:** Vipp1/IM30, PspA, ESCRT-III, membrane remodeling, cryoelectron microscopy, ring structure, cytoskeleton, LUCA, evolution, eukaryogenesis

## Abstract

Membrane remodeling and repair are essential for all cells. Proteins that perform these functions include Vipp1/IM30 in photosynthetic plastids, PspA in bacteria, and ESCRT-III in eukaryotes. Here, using a combination of evolutionary and structural analyses, we show that these protein families are homologous and share a common ancient evolutionary origin that likely predates the last universal common ancestor. This homology is evident in cryo-electron microscopy structures of Vipp1 rings from the cyanobacterium *Nostoc punctiforme* presented over a range of symmetries. Each ring is assembled from rungs that stack and progressively tilt to form dome-shaped curvature. Assembly is facilitated by hinges in the Vipp1 monomer, similar to those in ESCRT-III proteins, which allow the formation of flexible polymers. Rings have an inner lumen that is able to bind and deform membranes. Collectively, these data suggest conserved mechanistic principles that underlie Vipp1, PspA, and ESCRT-III-dependent membrane remodeling across all domains of life.

## Highlights

• PspA, Vipp1, and ESCRT-III are members of the same polymer-forming protein family
• ESCRT-III-like polymers function to remodel membranes across all domains of life
• Hinges in the Vipp1 monomer enable it to form a diverse set of flexible polymers
• The inner lumen of the dome-shaped Vipp1 ring binds and deforms membranes

## Graphical abstract

**Figure:**
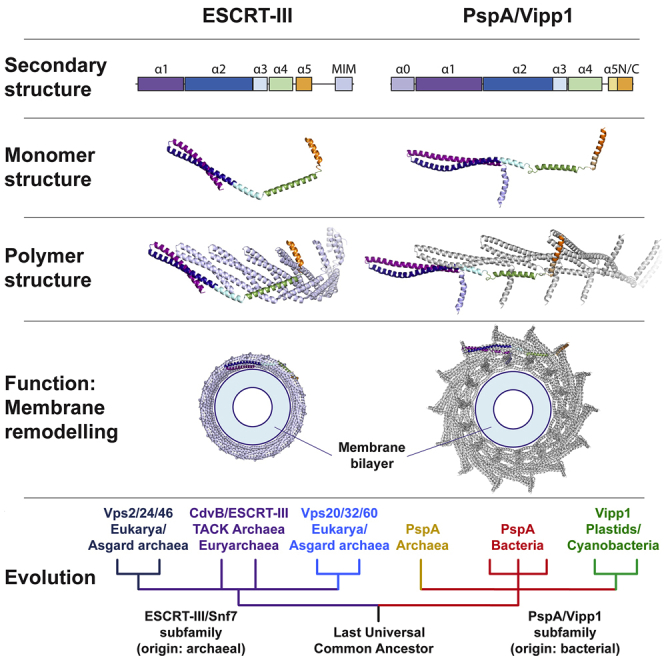

## Teaser

Phylogenetic and structural characterization of Vipp1 and PspA identifies them as ESCRT-III homologs and part of an ancient family of protein polymers that remodel membranes across the domains of life.

## Introduction

Various cytoskeletal elements, filaments, and membrane remodeling systems that were once viewed as the defining features of eukaryotic cells are now known to have a prokaryotic ancestry. Bacteria and some archaea have their own versions of tubulin (FtsZ), actin (MreB, FtsA, and ParM), intermediate filaments (Crescentin), and dynamins (BDLPs) (Bohuszewicz et al., 2016; Low and Löwe, 2006; Wagstaff and Löwe, 2018). These proteins perform functions in prokaryotes that are often analogous to their eukaryotic counterparts including cell division, cell shape control, and membrane remodeling. What, though, about ESCRT-III proteins? Until recently, this major class of protein polymers was only known in eukaryotes where they form composite polymers (Bertin et al., 2020; McCullough et al., 2018; Nguyen et al., 2020). ESCRT-III proteins are involved in multiple cell biological processes including multivesicular body formation, cytokinetic abscission, plasma membrane repair, nuclear envelope reformation, and viral budding (McCullough et al., 2018; Vietri et al., 2020). The idea that these proteins were specific to eukaryotes changed with the discovery of ESCRT-III homologs in TACK archaea (Lindås et al., 2008; Samson et al., 2008), where they have been implicated in membrane scission during viral budding (Liu et al., 2017), extracellular vesicle formation (Ellen et al., 2009), and cell division (Tarrason Risa et al., 2020). This, and the identification of even closer homologs of ESCRT-III proteins (and their regulators) encoded by the genomes of Asgard archaea, led to the suggestion that these eukaryotic signature proteins have an archaeal origin (Spang et al., 2015).

A unique feature of ESCRT-III polymers is their structural flexibility (Nguyen et al., 2020), enabling them to form helical tubes, rings, spirals, filaments, cones (Chiaruttini and Roux, 2017), and an array of structurally distinct composite polymers (Huber et al., 2020; McCullough et al., 2015; Nguyen et al., 2020). As examples of this, the X-ray structure of Snf7 showed how an ESCRT-III protein packs to build a linear proto-filament (Tang et al., 2015), while the cryoelectron microscopy (cryo-EM) structures of Vps24, and CHMP1B and IST1 co-polymers provided glimpses into the mechanisms by which ESCRT-III proteins self-assemble to form helical filaments with radically different lattices (Huber et al., 2020; McCullough et al., 2015; Nguyen et al., 2020). Because ESCRT-III polymers are implicated in the scission of membrane tubes from the inside during a wide array of cell biological processes, it is generally thought that they preferentially interact with negatively curved membranes. However, recent studies indicate that ESCRT-III proteins can also act on positively curved membranes *in vitro* (Bertin et al., 2020; McCullough et al., 2015; Nguyen et al., 2020) and possibly *in vivo* (Allison et al., 2013; Chang et al., 2019; Mast et al., 2018). When acting on membranes with either topology (Harker-Kirschneck et al., 2019), the AAA-ATPase Vps4 is thought to provide the energy required for membrane remodeling by driving stepwise changes in the composition and structure of ESCRT-III co-polymers (Harker-Kirschneck et al., 2019; Pfitzner et al., 2020).

Here, we identify PspA and Vipp1 proteins as members of the ESCRT-III superfamily. This is striking because these proteins share a common function in membrane remodeling. Thus, PspA functions in membrane stress response and repair in bacteria (Brissette et al., 1990; Joly et al., 2010; Kobayashi et al., 2007; McDonald et al., 2015; Yamaguchi et al., 2010), whereas Vipp1 functions in thylakoid membrane biogenesis and repair in cyanobacteria and chloroplasts (Aseeva et al., 2007; Fuhrmann et al., 2009b; Gao and Xu, 2009; Gutu et al., 2018; Kroll et al., 2001; Lo and Theg, 2012; Nordhues et al., 2012; Walter et al., 2015; Westphal et al., 2001; Zhang and Sakamoto, 2015). Like their ESCRT-III counterparts in eukaryotes, PspA and Vipp1 bind membranes and self-assemble as polymeric rings and tubes (Aseeva et al., 2004; Fuhrmann et al., 2009a; Liu et al., 2007). Until now, only a partial structure of PspA (Osadnik et al., 2015) and low resolution reconstructions of Vipp1 and PspA rings (Hankamer et al., 2004; Saur et al., 2017) had been determined, so that their mode of self-assembly and membrane binding remained obscure. In this study, we use cryo-EM to show that Vipp1 and PspA form ESCRT-III-like filaments and provide a mechanism for Vipp1-mediated membrane remodeling. This is in line with parallel work carried out by other groups (Gupta et al., 2021; Junglas et al., 2021, this issue of *Cell*). Collectively, our data show that the wider ESCRT-III family of polymers, which includes Vipp1 and PspA, arose prior to the divergence of bacteria and archaea over 3 billion years ago and play common, conserved roles in membrane remodeling and repair across all domains of life.

## Results

### PspA, Vipp1, and ESCRT-III are homologous

**Figure S1:**
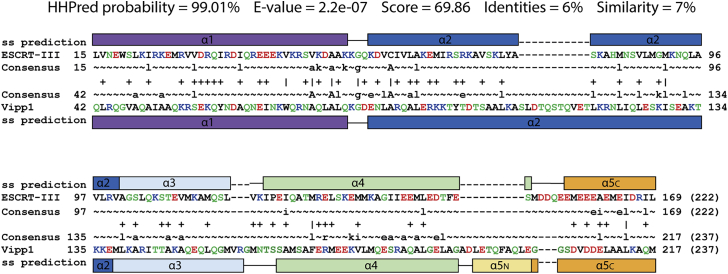
Evolutionary relationship of ESCRT-III/Snf7/CdvB and PspA/Vipp1 proteins, related to Figure 1 ESCRT-III/Snf7/CdvB and PspA/Vipp1 protein families are homologs based on an analysis performed using HHPred (Zimmermann et al., 2018); as indicated by HHPred probability, E-value and Score. The alignment of human CHMP3 (ESCRT-III) and *N. punctiforme* Vipp1 is based on their HMM profiles. Only highly conserved (uppercase) and moderately conserved (lowercase) HMM consensus positions are displayed. Vertical lines and plus signs indicate identical and similar HMM positions, respectively. CHMP3 and Vipp1 residues are color-coded according to their chemical properties: polar – green; positively charged – blue; negatively charged – red; and hydrophobic - black. Secondary structure (ss) predictions are shown with helices numbered based on both CHMP3 (PDB: 3FRT) and CHMP1B (PDB: 3JC1) structures.

**Figure 1:**
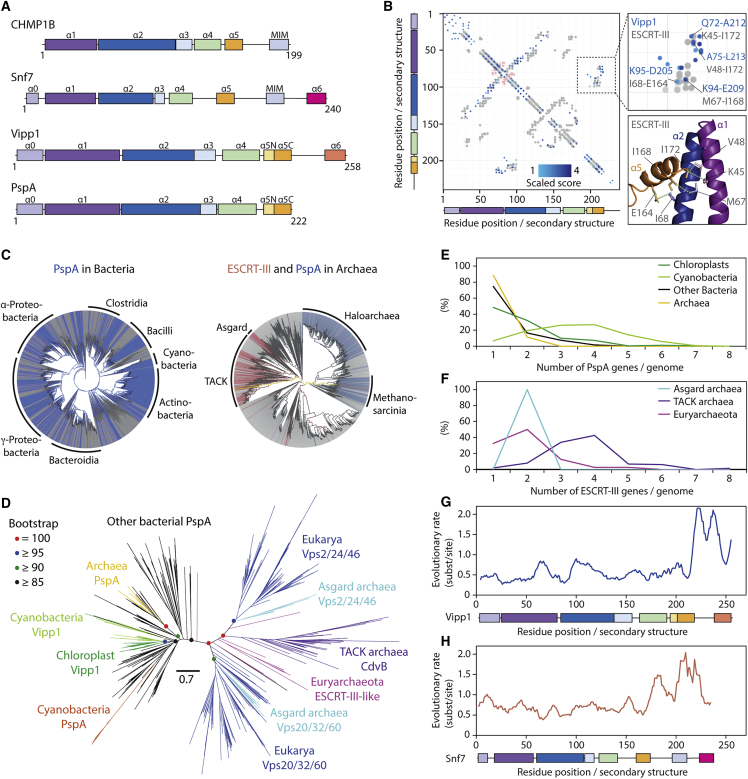
ESCRT-III/Snf7/CdvB and PspA/Vipp1 proteins are part of the same superfamily (A) ESCRT-III (human CHMP1B and yeast Snf7) and PspA (*N. punctiforme* Vipp1 and *E. coli* PspA) protein families are homologs and have a similar secondary structural organization (alpha helices are labeled α0–α6). Helices α6 have different colors because it is not clear if they are homologous across the PspA/Vipp1 and ESCRT-III/Snf7 families. (B) A co-evolutionary analysis reveals similarities in the tertiary structure of PspA and ESCRT-III proteins. Left: plot shows a co-evolutionary residue contact map (using numbering based on the *N. punctiforme* Vipp1 sequence) super-imposed on ESCRT-III residue-residue distance data extracted from the experimentally determined structure of human CHMP3 (PDB: 3FRT). Extent of blue shading represents the strength in covariance between Vipp1 co-evolving residue pairs. Grey and red circles indicate intramolecular and intermolecular contacts (<5 Å) in the CHMP3 X-ray crystal structure, respectively. Top right: inset focuses on selected Vipp1 evolutionary coupled residues in blue clustering with known contacts derived from CHMP3 in gray. Bottom right: these contacts are mapped onto the CHMP3 structure. (C) A phylogenetic analysis reveals a broad distribution of PspA/Vipp1 (blue) and ESCRT-III (red) homologs across bacteria (left, over 27,000 genomes) and archaea (right, over 1,500 genomes). Only a very few genomes encode both proteins (yellow). Genomes lacking both PspA/Vipp1 and ESCRT-III are presented in gray. (D) Tree of the PspA/ESCRT-III superfamily colored according to phylogenetic groups inferred under the best-fitting LG+C30+G+F substitution model. A long branch separates the PspA/Vipp1 (left) and ESCRT-III (right) subfamilies. Scale bar represents expected substitutions per site. (E and F) Number of copies of PspA/Vipp1 (top) and ESCRT-III/Snf7/CdvB (bottom) genes found per genome in different taxonomic groups. The analysis of the number of PspA in bacteria excludes cyanobacterial genes. (G and H) Site-specific evolutionary rates in units of expected number of substitutions per site across PspA/Vipp1 and ESCRT-III/Snf7 protein families using *N. punctiforme* Vipp1 (top) and yeast Snf7 (bottom) as reference sequences. See also Figure S1 and Data S1.

To identify other as yet unknown ESCRT-III relatives, we used sensitive protein sequence searches based on Hidden Markov Models (Zimmermann et al., 2018). Using eukaryotic ESCRT-III proteins as search queries, PspA and Vipp1/IM30 families were identified as bacterial ESCRT-III homologs with high statistical support (Figure S1; STAR Methods). In addition to similarity in primary sequence, PspA, Vipp1, and ESCRT-III families were also found to share similar secondary structural elements with five core helices α1–α5. In addition, these proteins often possess an N-terminal helix α0 implicated in membrane binding (Buchkovich et al., 2013; McDonald et al., 2017; Otters et al., 2013) and a C-terminal extension termed helix α6 (Figure 1A).

To probe their tertiary structure, we used a co-evolutionary analysis (Anishchenko et al., 2017; Ovchinnikov et al., 2014). This identified high-scoring residues that co-vary in PspA/Vipp1 evolution within a key conserved ESCRT-III interface that forms between helix α5 and helices α1 and α2 (Figure 1B). For ESCRT-III, this tertiary and quaternary interaction functions both to maintain the protein in its auto-inhibited closed monomeric conformation (Bajorek et al., 2009) and to stabilize the open polymeric form following a helix α5 domain-swap between subunits (McCullough et al., 2015). Thus, these data suggest that Vipp1 polymers may rely on similar contacts for their structural stability (Figure 1B).

We next looked at the wider evolution of the ESCRT-III/PspA superfamily. By mapping the presence or absence of different ESCRT-III homologs across the domains of life, it became clear that PspA/Vipp1 proteins are widely distributed across bacteria (Figure 1C). Conversely, CdvB/Chmp/Snf7-type proteins are found in all eukaryotes and in clusters of archaea, largely within Asgard and TACK superphyla (Spang et al., 2015; Figure 1C). Moreover, this analysis identified a few additional CdvB/Chmp/Snf7-type ESCRT-III homologs in members of the Euryarchaeota, as previously reported (Makarova et al., 2010). Finally, a number of PspA/Vipp1-type homologs were found within the euryarchaeal lineages Haloarchaea and Methanosarcinales (Figure 1C).

A phylogenetic analysis of the PspA, Vipp1, and ESCRT-III superfamily as a whole revealed a long branch separating the bacterial and archaeal/eukaryotic clades, with euryarchaeal ESCRT-III homologs branching near the base of the ESCRT-III subtree. The PspA/Vipp1 homologs found in Haloarchaea and Methanosarcinales are nested within the bacterial PspA clade and therefore appear to be relatively recent acquisitions by horizontal gene transfer (HGT). Because CdvB/Chmp/Snf7-type proteins are widely distributed across archaea and eukaryotes, and PspA/Vipp1-type proteins are widely distributed across bacteria, this phylogeny (Figure 1D; Data S1A and S1B) is consistent with the hypothesis that a single gene ancestral to PspA, Vipp1, and ESCRT-III was already present in the last universal common ancestor (LUCA).

Our evolutionary analysis also confirmed that Vipp1 likely arose from a *pspA* gene duplication (Westphal et al., 2001) and is widely distributed among cyanobacteria and eukaryotic plastids derived from cyanobacteria. Interestingly, although most bacteria and archaea possess a single copy of PspA, Vipp1 tends to exist in multiple copies in cyanobacterial genomes (Figure 1E). This is a common feature of eukaryotic ESCRT-III proteins (Leung et al., 2008), which often form composite polymers in both TACK archaea (Pulschen et al., 2020; Tarrason Risa et al., 2020) and eukaryotes (Bertin et al., 2020; Nguyen et al., 2020; Pfitzner et al., 2020). In addition, Euryarchaeota and Asgard archaea genomes encode multiple copies of ESCRT-III proteins (Figure 1F).

Finally, we mapped rate of evolutionary variance across both ESCRT-III and PspA family proteins using yeast Snf7 and *Nostoc punctiforme* Vipp1 as reference sequences. This analysis showed that both families share a similar evolutionary profile (Figures 1G and 1H) with well-conserved core secondary structure including the interface between helix α5 and helices α1 and α2 (Figure 1B). Because the regions C-terminal to helix α5 evolved much more rapidly, we were unable to detect homology between Snf7 and Vipp1. Thus, it is uncertain whether the common helical elements within the C termini of both families are homologous or the products of convergent evolution. This includes the MIM domain, which in archaeal and eukaryotic ESCRT-III proteins physically associates with the AAA-ATPase Vps4 (McCullough et al., 2018).

Overall, our evolutionary analyses show that PspA and Vipp1 are members of the ESCRT-III superfamily. To investigate whether this evolutionary conservation extends to structural similarities between the bacterial and eukaryotic members of the family, such as CHMP1B/IST1 and Snf7, we proceeded to carry out cryo-EM studies on a member of the bacterial branch of this ESCRT-III superfamily: *N. punctiforme* Vipp1.

### Vipp1 purification and cryo-EM

**Figure S2:**
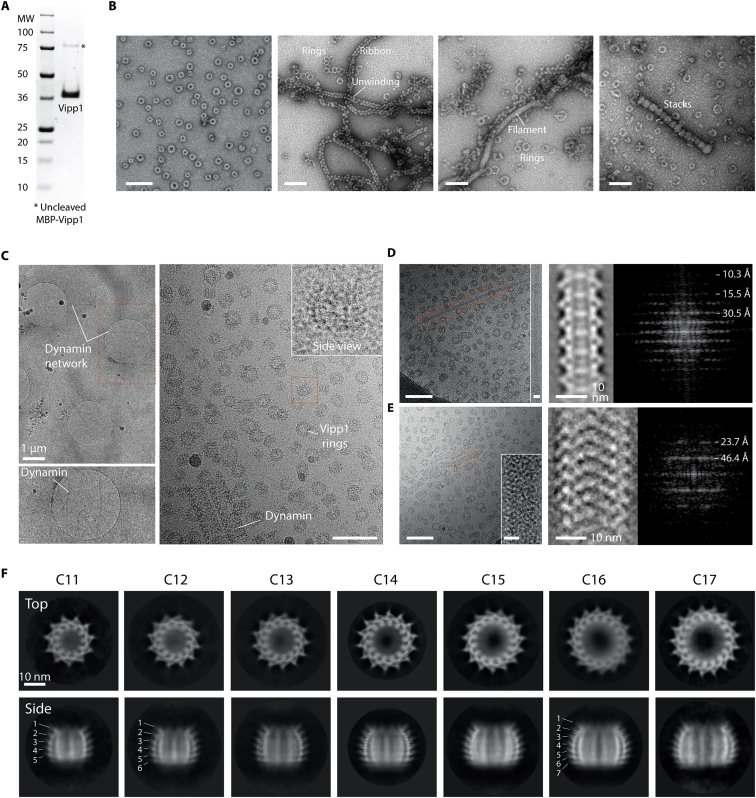
Purification and electron microscopy of *N. punctiforme* Vipp1, related to Figure 2 (A) SDS-PAGE showing purified Vipp1. Note that Vipp1 migrates at ~38 kDa whereas its true mass is 28.7 kDa. (B) Gallery of negative stain EM micrographs showing Vipp1 forming rings, helical ribbons, filaments and stacks. Scale bar = 100 nm. (C) Cryo-EM micrographs showing Vipp1 rings mixed with human Dynamin 1 filaments. Left panels- low magnification overview of the holey grid showing the Dynamin 1 filament network used to maintain ice thickness and promote Vipp1 side views. Red rectangle outlines zoom panel below. Right panel- example micrograph showing Vipp1 rings including side views together with the Dynamin 1 filaments. Scale bar = 100 nm. (D) Vipp1 filaments were sometimes observed among Vipp1 rings. The cryo-EM micrograph shows an example of a 14 nm diameter Vipp1 filament (left) with associated class average (middle) and Fourier Bessel analysis (right). The filament shows a helical repeat at 30.5 Å and 15.5 Å. 30.5 Å is consistent with the axial rise between the hairpin motif of neighboring rungs. Scale bar = 100 nm (left) and 10 nm (left inset). (E) The cryo-EM micrograph shows an example of a 24 nm diameter Vipp1 filament (left) with associated class average (middle) and Fourier Bessel analysis (right). Scale bar = 100 nm (left) and 10 nm (left inset). The filament shows a helical repeat at 46.4 Å and 23.7 Å. (F) Gallery of Vipp1_C11-C17_ 2D class averages from the cryo-EM dataset showing end and side views.

For the structural analysis, full-length Vipp1/IM30 (amino acids 1–258, 28.7 kDa) from *N. punctiforme* was expressed in *Escherichia coli* with an N-terminal maltose-binding protein (MBP) fusion, purified by amylose affinity chromatography and then separated from MBP by TEV cleavage and size exclusion chromatography (Figure S2A). Negative stain (NS) EM analysis of Vipp1 revealed a remarkable array of polymeric assemblies (Figure S2B), including rings (Figure S2C), ring stacks, filaments (Figures S2D and S2E), and ribbons reminiscent of Vipp1 in other systems (Aseeva et al., 2004; Fuhrmann et al., 2009a; Hennig et al., 2017; Theis et al., 2019). For this study, we focused on Vipp1 rings because they have been widely reported from cyanobacteria through to plants (Aseeva et al., 2004; Fuhrmann et al., 2009a; Liu et al., 2007) and appear to constitute a reproducible structural form for this class of proteins. In order to determine their architecture, the sample was flash frozen on holey carbon grids for cryo-EM analysis. Because micrographs of Vipp1 in thin vitreous ice revealed preferential orientation bias, we mixed Vipp1 with pre-formed rat Dynamin 1 filaments before vitrification (Sundborger et al., 2014) to thicken the ice layer around the Vipp1 particles to capture a wider array of side views (Figure S2C). Ultimately, 2D class averages of the Vipp1 rings revealed a spectrum of seven symmetries ranging from C11 to C17 (Figure S2F).

### The Vipp1 monomer has an ESCRT-III-like fold

**Figure S3:**
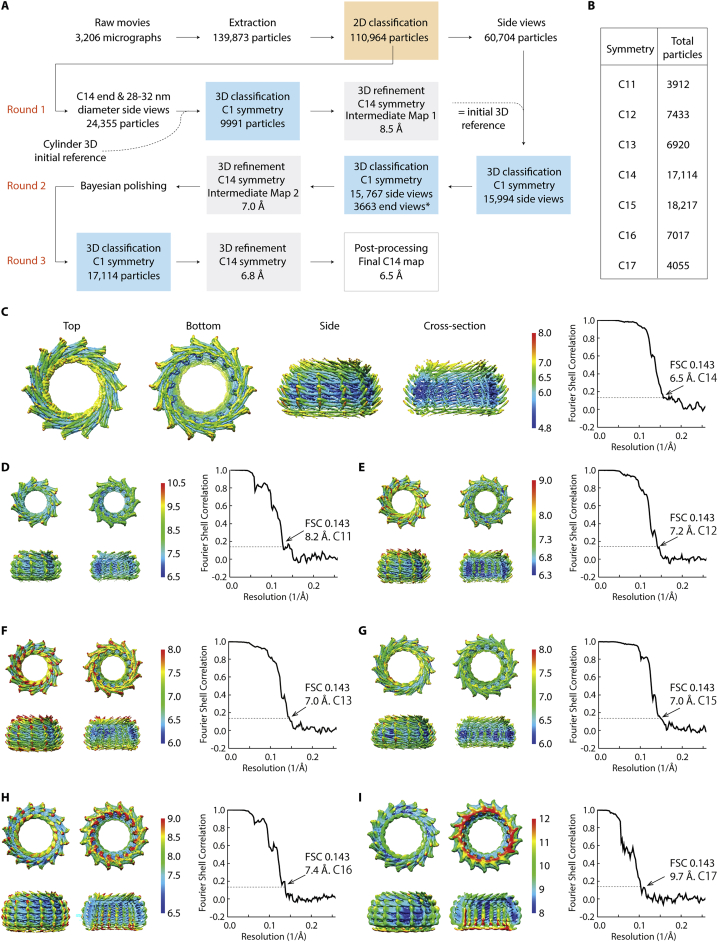
Vipp1_C11–C17_ processing strategy with local resolution maps and FSC curves, related to Figure 2 (A) Vipp1 C14 processing strategy for 3D reconstruction and refinement. ^∗^ End views derived from 2D classification (orange box). (B) Table showing total particle number included in final 3D refinements for each Vipp1 ring symmetry. (C-I) Gallery of sharpened maps contoured between 4-6σ showing local resolution estimates and associated gold standard FSC curves. In Vipp1_C14_, 4.8 Å resolution was reached around the ring equator.

**Figure S4:**
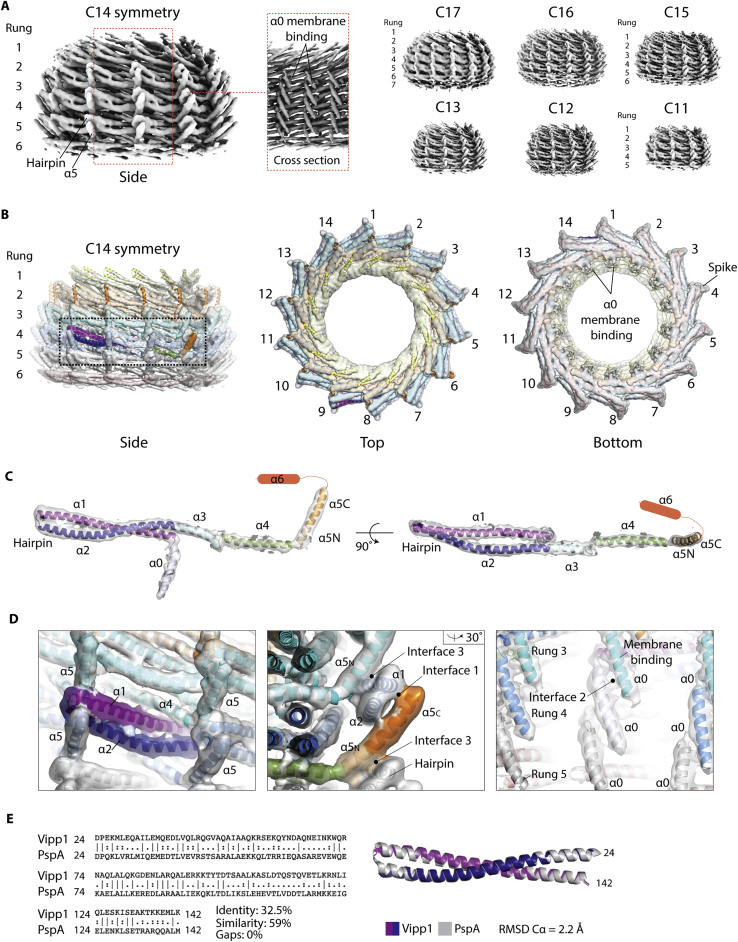
Vipp1_C11–C17_ map quality and model building, related to Figure 2 (A) Gallery of Vipp1_C11-C17_ EM density maps contoured between 4-6σ. (B) Vipp1_C14_ map fitted with 84 Vipp1 subunits. Subunits in rungs 1 and 6 were partially built with helices α4, α5 and α6 omitted due to disordered or absent density. Density for helix α6 was never observed. Map contoured at 4σ. (C) Isolated Vipp1 monomer showing quality of map, build and fit. The subunit extracted is indicated in the rectangular box in (B). (D) Select regions of Vipp1_C14_ showing quality of map, build and fit. Left panel- hairpin motif. Middle panel- end view of a hairpin motif forming both intra-rung Interface 1 and inter-rung Interface 3 with helix α5. Right panel- helix α0 stacking forms Interface 2. (E) Left panel- pairwise alignment of *N. punctiforme* Vipp1 (Uniprot code B2J6D9) with *E. coli* PspA (Uniprot code P0AFM6) amino acids 24-142 (hairpin motif). Right panel- superposition of Vipp1_C14_ amino acids 24-142 (rung number 4) with PspA partial structure (PDB: 4WHE). RMSD = relative mean square deviation.

**Figure 2:**
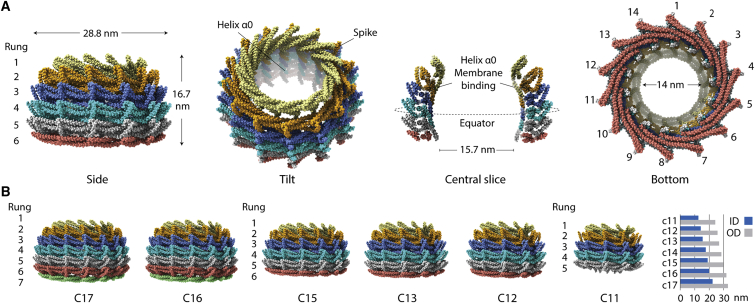
Main chain structures of Vipp1_C11–C17_ rings (A) Structure of Vipp1_C14_. The ring comprises a stack of six rungs. Dome-shaped curvature is observed from the side and in the central slice. The ring is widest at the equatorial plane both internally and externally. (B) Side view gallery of Vipp1_C11–C17_ rings. Bar chart indicates maximal outer diameter (OD) and maximal inner diameter (ID) as measured at the external and internal equator. Note that Vipp1_C16–C17_ have seven rungs whereas Vipp1_C11_ has just five. See also Figure S2, Figure S3, Figure S4, Figure S5 and Table S1.

**Figure S5:**
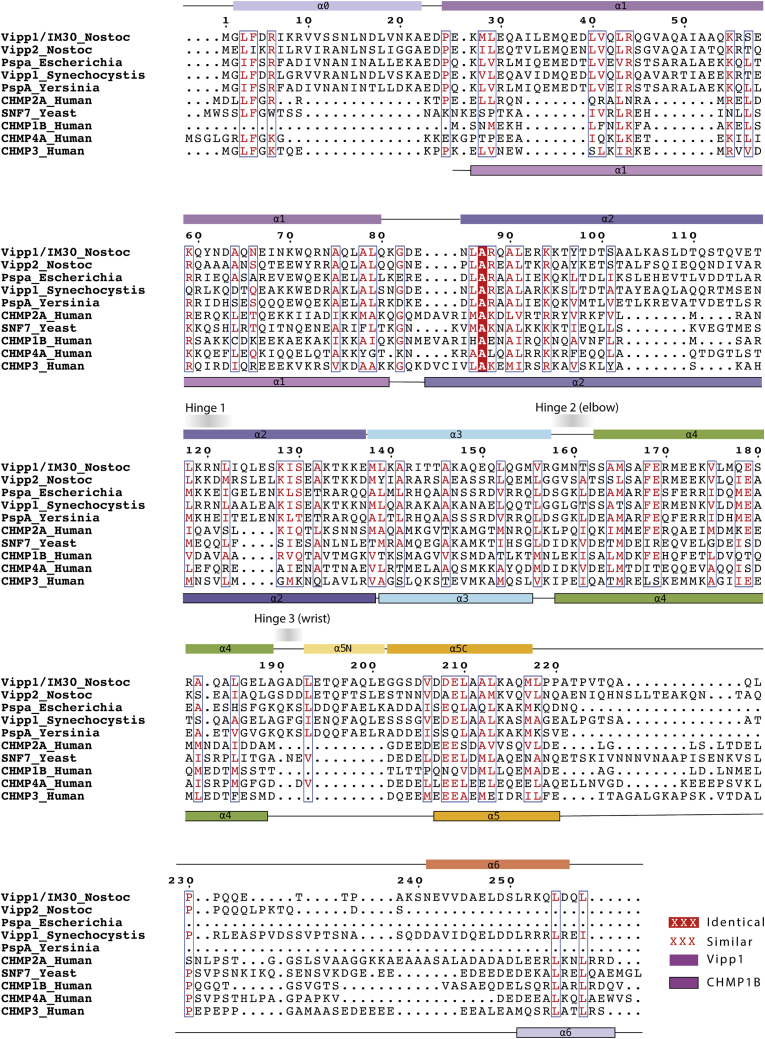
Vipp1 secondary structure assignment and sequence alignment with PspA and ESCRT-III, related to Figure 2 Sequences were aligned using Clustal Omega and include Vipp1/IM30 *Nostoc punctiforme* (Uniprot code B2J6D9), Vipp2 *Nostoc punctiforme* (Uniprot code B2J6E0), *Escherichia coli* PspA (Uniprot code P0AFM6), *Synechocystis sp*. Vipp1 (Uniprot code A0A068MW27), *Yersinia pestis* PspA (Uniprot code Q0WEH0), human CHMP2A (Uniprot code O43633), yeast SNF7 (Uniprot code P39929), human CHMP1B (Uniprot code Q7LBR1), human CHMP4A (Uniprot code Q9BY43) and human CHMP3 (Uniprot code Q0Y3E7). Cartoon shows Vipp1 (top) and CHMP1B (bottom, PDB: 6TZ4) secondary structure.

By reconstructing the C14 symmetry ring (Vipp1_C14_), we achieved an overall resolution of 6.5 Å with highest resolution regions reaching 4.8 Å (Figures S3 and S4A; Table S1). This was sufficient to allow unambiguous assignment of the helical domains within the Vipp1 monomer and asymmetric unit and consequently the entire 2.4 MDa Vipp1_C14_ ring containing 84 subunits (Figures 2 and S4B–S4D; STAR Methods). To build the Vipp1_C14_ monomer, a homology model was initially generated from the PspA crystal structure hairpin motif (aa 24–142). For these amino acids, PspA and Vipp1 share 32.5% sequence identity and 58% similarity with zero gaps, which indicates a conserved sequence register in this section (Figure S4, Figure S5E and S5). The Vipp1 hairpin homology model with its distinct axial twist closely fitted the Vipp1_C14_ map and acted as an anchor from which to build the remaining main chain. Reconstructions were also generated for the other six symmetries and complete ring models built. Sizes ranged from the 1.6 MDa Vipp1_C11_ ring with 55 subunits to the 3.4 MDa Vipp1_C17_ ring with 119 subunits.

**Figure 3:**
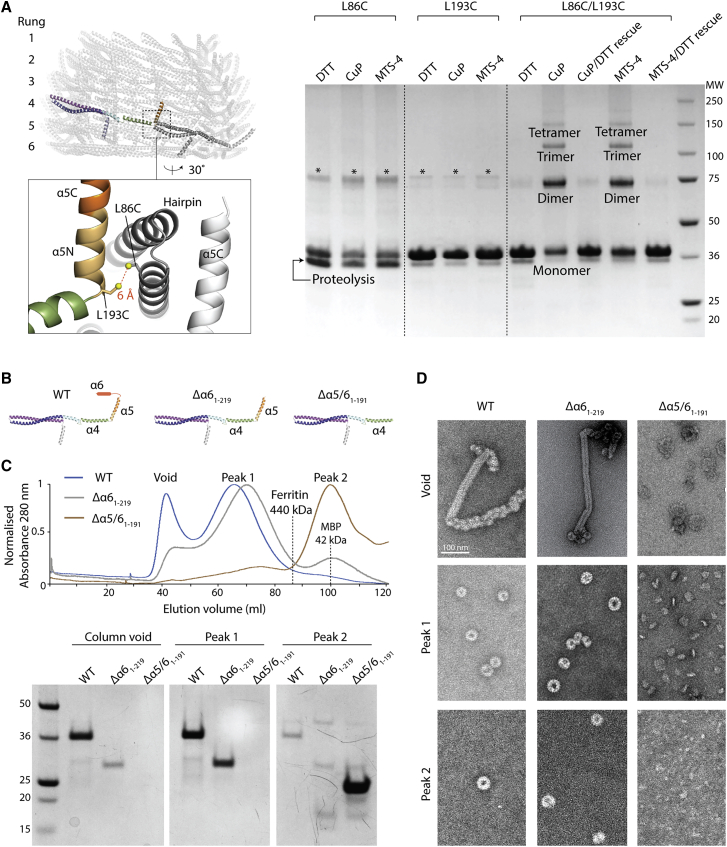
Vipp1 structure validation and polymer assembly (A) Vipp1 monomer structure sequence register validation based on cysteine cross-linking. Zoom box indicates position of L193C and L86C mutants (Vipp1_L86C/L193C_) with the sulfur atoms predicted to be ∼6 Å within the Vipp1_C14_ structure. Vipp1_L86C/L193C_ forms an inter-rung connection between helix α5 and the hairpin motif (Interface 3). Disulfide bond formation is observed only in the presence of oxidizing agent Cu(II)-phenanthroline (CuP) and MTS4 cross-linker. Disulfide bond formation can be rescued upon subsequent DTT incubation. ^∗^Indicates uncleaved MBP-Vipp1 fusion protein. Vipp1_L86C_ showed higher levels of proteolysis and resistance to full MBP cleavage than normal, presumably due to the sensitive position of the mutation. (B) Vipp1 subunit cartoon schematics showing wild-type (WT), Vipp1Δα6_1–219_, and Vipp1Δα5/6_1–191_ secondary structure. (C and D) Filament assembly assay. In (C), comparison of WT, Vipp1Δα6_1–219_, and Vipp1Δα5/6_1–191_ size exclusion profiles using a sephacryl S-500 resin. Associated SDS-PAGE is shown analyzing the column exclusion limit (void volume), Peak 1 containing high molecular weight species greater than Ferritin (440 kDa) and usually associated with Vipp1_C11–C17_ rings, and peak 2 associated with low molecular weight proteins such as maltose-binding protein (42 kDa). Negative stain EM analysis is shown in (D) for void volume, peak 1, and peak 2. WT and Vipp1Δα6_1–219_ behave similarly, with filaments observed in the void volume and Vipp1_C11–C17_ rings observed in peak 1. In contrast, Vipp1Δα5/6_1–191_ does not form filaments or rings and instead runs as a low molecular weight species consistent with monomer or dimer. This experiment shows that helix α5 is essential for polymer formation while helix α6 is not. It also broadly validates the helix α5 and α6 domain assignment within the Vipp1 structures.

In order to validate these Vipp1_C11–C17_ structures, the accuracy of the sequence register within the Vipp1 monomer was tested by cross-linking cysteine residues at opposing ends of the Vipp1 monomer (Vipp1_L86C/L193C_ and Figure 3A). Consistent with Vipp1_C11–C17_ structures, which position these residues at an inter-subunit contact with the cysteine sulfur atoms predicted ∼6 Å apart, clear dithiothreitol (DTT)-sensitive band shifts were observed for Vipp1_L86C/L193C_ in the presence of the oxidizing agent ortho-Cu(II)-phenanthroline (CuP) or the cross-linker MTS4 with a 7.8 Å span. Importantly, no equivalent band shifts were observed for the single cysteine mutants Vipp1_L86C_ or Vipp1_L193C_.

**Figure 4:**
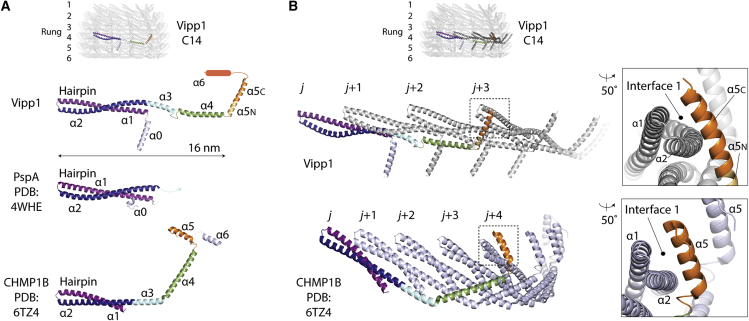
Vipp1 has a similar fold and assembly mechanism as ESCRT-III (A) Vipp1 has the same helical domain organization as ESCRT-III proteins, such as CHMP1B. The hairpin motif is a hallmark of the Vipp1, PspA, and ESCRT-III families. (B) Vipp1 and CHMP1B form similar polymers based on hairpin packing and helix α5 domain swap. A single circular polymer forms each rung of the Vipp1 ring. Zoom boxes show Interface 1 where the helix α5 domain swap binds the hairpin tip of a neighboring subunit in both Vipp1 (*j*+3) and CHMP1B (*j*+4).

In order to compare the structures of Vipp1 and ESCRT-III proteins, we followed the ESCRT-III nomenclature (Figure S5; McCullough et al., 2015) and used CHMP1B and other available structures as guides (Bajorek et al., 2009; Huber et al., 2020; Nguyen et al., 2020; Tang et al., 2015). The hairpin motif, which is formed by helix α1 and conjoined helices α2/α3, was observed to be a conserved hallmark of Vipp1, PspA, and ESCRT-III proteins (Figure 4A). In addition, helix α4 was separated from hairpin helices α2/α3 by a linker region that corresponded to the ESCRT-III elbow (McCullough et al., 2015). Helix α5 was then angled from helix α4 in both Vipp1 and ESCRT-III proteins. In addition to these core common helices α1–α5 between Vipp1, CHMP1B, and other ESCRT-III polymers, the Vipp1 monomer includes an N-terminal helix α0 (aa 1–22) that extends perpendicular to the hairpin and mediates membrane binding in both PspA and Vipp1 systems (Heidrich et al., 2016; McDonald et al., 2015, 2017; Otters et al., 2013). Helix α0 is not observed in CHMP1B, but it is shared by ESCRT-III proteins such as Vps2/CHMP2, Vps24/CHMP3, and Snf7/CHMP4, where it is reported to also mediate membrane binding (Figures 1A and S5; Buchkovich et al., 2013). Vipp1 is distinguished from PspA by an additional C-terminal extension (aa 220–258) comprising a flexible linker and predicted helix α6 (Zhang et al., 2016). Although the C-terminal extension has been shown to negatively regulate Vipp1 self-association *in vivo* (Zhang et al., 2016) and might constitute a second lipid binding domain capable of modulating membrane fusion (Hennig et al., 2017; 2015), it did not yield a density in the Vipp1_C14_ map, suggesting that this region is highly flexible (Figures 4A and S4). The same is true for C-terminal extensions in ESCRT-III proteins, which serve as flexible regulatory and/or protein-protein interaction domains (McCullough et al., 2018).

### Vipp1 rungs form ESCRT-III-like filaments

Vipp1_C14_ rings are assembled from six rungs stacked on top of each other (Figure 2A). Each rung differs in conformation so that an asymmetric dome-shaped curvature is achieved when rings are viewed from the side. Rungs are maximally constricted at the top and bottom with internal lumen diameters of 14 nm and 15.7 nm, and widest at the ring equator between rungs 3 and 4 with an internal diameter of 17.5 nm (Figures 2A and 2B). This unique asymmetric curvature is a feature of all Vipp1_C11–C17_ ring symmetries (Figure 2B).

Within each rung, Vipp1 subunits form a staggered polymer with subunit *j* contacting neighboring subunits *j*+1 and *j*+3 (Figure 4B). Hairpin motifs pack side by side so that helices α1, α2, and α3 of subunit *j* form an extended interface underneath the hairpin of neighboring subunit *j*+1. Concurrently, the helix α5 C terminus (α5C) of subunit *j* binds across the hairpin tip of subunit *j*+3 forming a contact termed here Interface 1. Importantly, similar hairpin stacking and the equivalent hairpin/helix α5 interface is observed within the CHMP1B filament between subunit *j* and subunit *j*+4 (Figure 4B; McCullough et al., 2015; Nguyen et al., 2020). The hairpin-helix α5 contact constitutes a domain swap in polymerized CHMP1B that is also observed in other ESCRT-III proteins (Huber et al., 2020). Interface 1 is conserved and was predicted for Vipp1 by our co-evolutionary and rate of variance analyses (Figures 1B, 1G, and S5). To probe the contribution of Interface 1 in polymer assembly, Vipp1 truncations were generated by removing either the helix α6 C-terminal extension (Vipp1Δα6_1–219_) or helices α5 and α6 (Vipp1Δα5/6_1–191_). Based on the Vipp1_C11–C17_ models, where helix α5C is positioned at the tip of the spike (Figure 2A), α6 helices likely coat the ring outside surface and do not contribute directly to polymer formation. Accordingly, both native Vipp1 and Vipp1Δα6_1–219_ formed rings and filaments as assayed using gel filtration and NS EM. By contrast, both ring and filament formation was abolished in the Vipp1Δα5/6_1–191_ mutant (Figure 3B-D). These results indicate that helix α5 and Interface 1, both of which are conserved in ESCRT-III systems, are essential for Vipp1 filament and ring assembly.

### Conserved hinge regions facilitate dome-shaped curvature within Vipp1 rings

**Figure 5:**
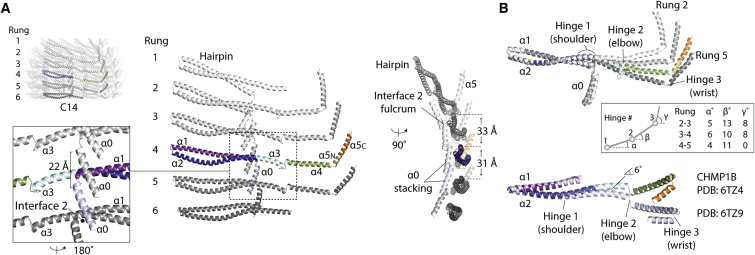
Analysis of the Vipp1_C14_ ring asymmetric unit (A) The ring asymmetric unit comprises an axial stack of six subunits each in its own distinct conformation. The position of helices α4–α6 in rungs 1 and 6 could not be assigned with certainty in the map so were omitted from the model. Zoom box shows Interface 2 formed from helix α0 contacting neighboring helix α0 and helix α1 N terminus. (B) Vipp1 and CHMP1B share conserved flexible joints called hinge 1–3 (shoulder, elbow, and wrist). Superposition of Vipp1 asymmetric unit subunits from rungs 2–5 aligned onto hairpin helices α1 and α2. Subunits transition between negatively and positively curved conformations from the top (rung 2) to the bottom (rung 5). See also Videos S1, S2, and S3.

The basic building block (or asymmetric unit) of Vipp1_C14_ is a stack of six monomers, each one located in a different rung, which when repeated forms a ring (Figure 5A). Intriguingly, the six subunits within each asymmetric unit have distinct conformations, which is made possible by three hinge regions that provide Vipp1 with conformational versatility (Figure 5B; Videos S1, S2, and S3). The hinges are located at the C terminus of helix α2 (hinge 1 or shoulder) between helix α3 and α4 (hinge 2 or elbow) and between helix α4 and α5 (hinge 3 or wrist). Within each ring, the flex in subunit structure is progressive, curling the ends of the asymmetric unit to give rise to the dome-shaped curvature. Importantly, similar subunit flexibility is seen in ESCRT-III, where the equivalent hinge regions are used to enable polymers to assume different filament lattices, curvatures, and to drive helical filament constriction (Nguyen et al., 2020; Huber et al., 2020). We note that hinge 1 has not previously been reported yet accounts for ∼6° of the observed flexibility in both Vipp1 and CHMP1B, albeit along a different axis (Figure 5B).

Video S1. Morph between superposed Vipp1 subunits within the Vipp1_C14_ asymmetric unit, related to Figure 5B Complete subunits from rungs 2-5 only are shown. Conformational change is mediated by Hinges 1-3 conserved between Vipp1 and ESCRT-III-like proteins.

Video S2. Front view morph between superposed Vipp1_C11-C17_ asymmetric units, related to Figure 5B Morph shows range of conformational changes between different ring symmetries.

Video S3. End view morph between superposed Vipp1_C11-C17_ asymmetric units, related to Figure 5B

### How rung stacking induces dome-shaped curvature

**Figure 6:**
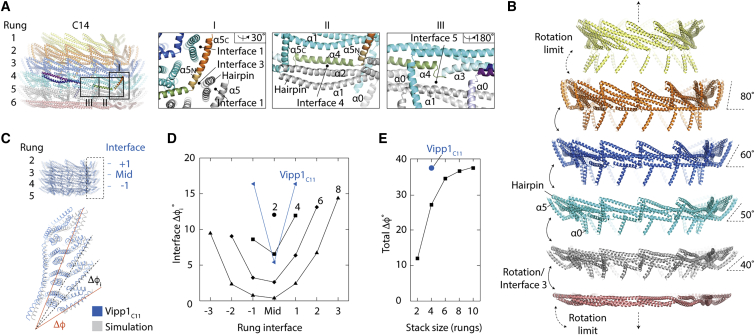
Mechanism for Vipp1 axial or dome-shaped curvature (A) Analysis of inter-rung contacts. Zoom boxes show Interfaces 3–5, which combined with Interface 2 (Figure 5A), define all inter-rung contacts. (B) Exploded side view of Vipp1_C14_ shows how each ring comprises a stack of discrete rungs. Each rung constitutes a circular Vipp1 polymer with a distinct conformation. Helix α5 is sandwiched between an intra-rung hairpin (Interface 1) and an inter-rung hairpin (Interface 3), which rotate collectively to induce ring constriction and curvature. Geometric constraint ultimately limits constriction. (C–E) MD simulations based on Vipp1_C11_ show that inter-rung interfaces define dome-shaped curvature. (C) Overlay of the Vipp1_C11_ structure with a four-rung simulation resulting in an equilibrium structure with dome-shaped curvature. Δφ_i_ = inter-rung rotation of helix α5. Δφ = cumulative rotation of helix α5 over all rungs. (D) Δφ_i_ plots from simulations with 2, 4, 6, and 8 rung stacks with C11 symmetry. Δφ_i_ for Vipp1_C11_ rungs 2–5 (blue). The largest rotations are observed at the ring top and bottom. (E) Δφ as a function of rung stack size. Geometric constraints limit the total Δφ to near 40° regardless of stack size. The limit in Δφ from simulated stacks matches the Δφ from Vipp1_C11_. See also Figure S6 and Video S4.

**Figure S6:**
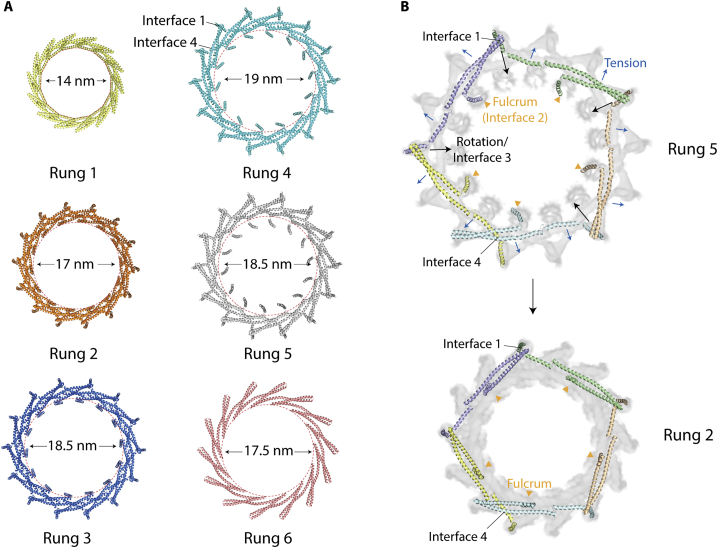
Mechanism for Vipp1 axial or dome-shaped curvature, related to Figure 6 (A) Exploded top view of Vipp1_C14_ shows how each ring comprises a stack of discrete rungs. Each rung constitutes a circular Vipp1 polymer with a distinct conformation. Ring diameters were measured at the helix α1 N terminus so as to highlight hairpin constriction between rungs. (B) Comparison of rung 5 and rung 2 to highlight the conformational changes required to drive dome-shaped curvature. For each rung only those subunits are shown (*j* and *j*+3) that interconnect via Interface 1 to form one turn. Polymer tilt involves hairpin and helix α5 rotation (Interface 1 and 3) around Interface 2, which appears to represent a fulcrum within the center of each subunit. Helix α0 within Interface 2 also rotates to create the dome-shaped curvature of the inner lumen. Increasing polymer or filament tilt and rotation builds subunit tension until ultimately geometric constraint limits the formation of Interface 3 and/or Interface 1 along with further rung stacking and constriction.

Within Vipp1 rings, each rung associates with its neighbor through just four interfaces. At the Vipp1 N terminus, helices α0 stack axially so that they line the ring lumen (termed Interface 2), where they are well positioned to bind membrane (Figures 2A, 5A, and S4A). As well as binding rungs together, Interface 2 also serves as a fulcrum mediating the sequential tilting of each helix α0 that defines the curvature of the ring lumen (Figure 5A). Interface 3 forms between the N terminus of helix α5 (α5N) and the hairpin tip from the rung below (Figure 6A). Although the amino acids implicated in Interface 3 are highly conserved in both Vipp1 and PspA family members, they are missing in ESCRT-III proteins, which do not possess an α5 N-terminal helical extension (Figure S5). The remaining inter-rung contacts, Interfaces 4 and 5, form smaller packing interfaces between helix α4 and helix α2 and between hinge 2 and helix α1, respectively (Figure 6A). As dome-shaped curvature increases toward the top of the ring, helix α5 rotates from being oriented at 40° relative to the horizontal in rung 5 (Figures 6B and S6A) to a maximum of 80° in rung 2. Due to Interfaces 1 and 3, the two hairpins bound to helix α5 co-rotate with the effect that they are pulled inward pivoting around Interface 2 (Figure S6B). This suggests the hypothesis that rung stacking causes lattice tension to build until a maximum bending limit is reached. At this point, additional bound subunits cannot flex sufficiently to form Interface 1 and/or 3, impeding further rung stacking.

### Modeling dome-shaped curvature in Vipp1 rings

**Figure S7:**
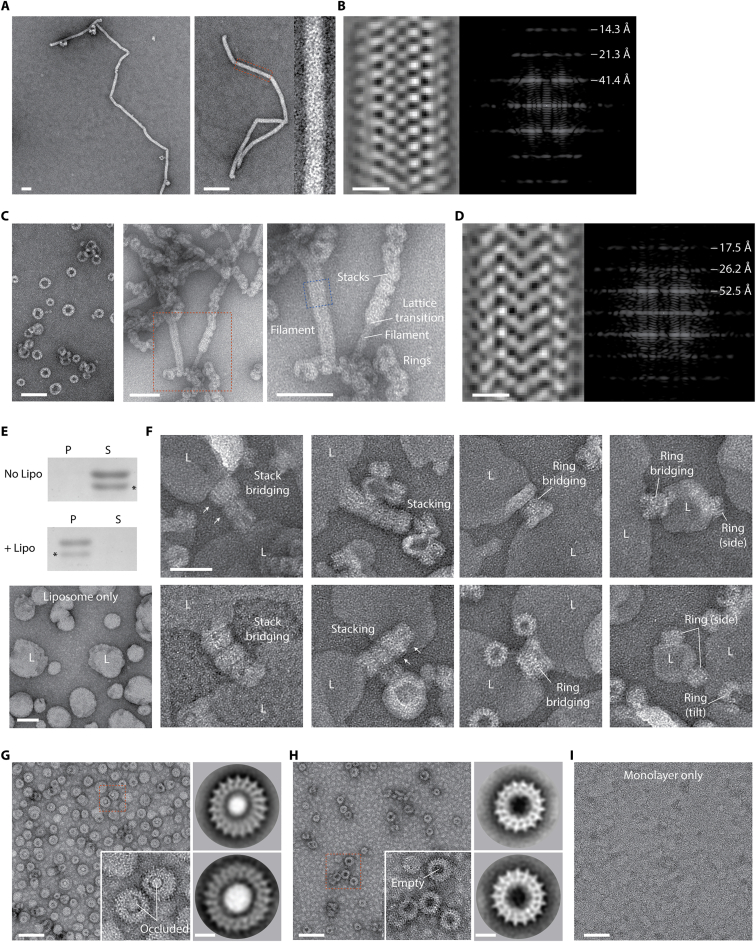
Negative stain EM analyses of Vipp1 and Vipp1 mutants in the presence and absence of lipids, related to Figure 7 (A) Vipp1_F197K/L200K_ forms unusually long filaments with broadly uniform diameter suggesting that loss of Interface 3 impedes filament tilt and axial curving. Zoom panel shows close up of the filament outlined by the dotted rectangle. Filament diameter is 25.5 nm nm. Scale bar = 50 nm (left) and 100 nm (right). (B) Vipp1_F197K/L200K_ filament class average with associated Fourier Bessel analysis. Scale bar = 10 nm. (C) Vipp1Δα6_1-219_ forms individual rings, ring stacks and filaments. Compared to native Vipp1, Vipp1Δα6_1-219_ has a significantly greater propensity to form ring stacks and filaments. Zoom panel shows region outlined by dotted red rectangle where ring stacks can be observed morphing into filaments and acting as nucleation points for filament formation and lattice transitions. Scale bar = 50 nm. (D) Vipp1Δα6_1-219_ filament (dotted blue box in (C) class average with associated Fourier Bessel analysis. Filament diameter is 27.5 nm. Scale bar = 10 nm. (E) Native Vipp1 spin assay in the presence and absence of *E. coli* liposomes. Vipp1 is observed in the pellet fraction only in the presence of the liposomes, indicative of lipid binding. ^∗^ indicates Vipp1 proteolysis. (F) Gallery of micrographs showing how Vipp1 rings decorate and tether liposomes (L) together. Individual rings or ring stacks form bridges between liposomes. Vipp1 is rarely observed unattached to a liposome and resting on the carbon surface. Vipp1 stacks may reduce in diameter to form cones (white arrows). Scale bar = 50 nm. (G) Left panel - Vipp1 rings decorate a lipid monolayer (ML). Scale bar = 100 nm. Zoom panel shows region outlined by dotted rectangle. Monolayer is drawn into the ring and occludes the lumen (white central density). Right panels- example class averages with scale bar = 10 nm. (H) In the absence of monolayers, Vipp1 rings have empty lumens (black central density). Right panels- example class averages with scale bars as in (G). (I) Monolayers only. Scale bar = 100 nm.

To test whether the dome-shaped curvature observed in Vipp1 rings depends on a combination of inter-rung contacts and geometric constraints, as suggested by these data, an idealized elastic-network model of the Vipp1_C11_ ring was constructed. First, an “average rung” was defined that represented the average shape of a single isolated Vipp1_C11_ rung. Then, we defined interactions between neighboring rungs based on the contacts between rungs 3 and 4 of the Vipp1_C11_ structure (Figures 2B and 6C). Finally, Vipp1 rings were initialized as cylindrical stacks of between 2 and 10 average rungs, which were then minimized subject to the elastic network (Video S4). The model rings formed Vipp1-like dome-shaped curvature with helix α5 rotation (Δϕ_i_) increasing with rung distance from the ring equator (Figure 6D). Strikingly, the cumulative helix α5 rotation (Δϕ) between all rung interfaces plateaued near 40°, indicating a strict geometrical bending limit. Remarkably, this maximal rotation within the simulated stacks closely matched the measured experimental Δϕ in Vipp1_C11_ (Figure 6E), as well as the cumulative ∼40° helix α5 rotations observed for Vipp1_C14_ (Figure 6B). Thus, the simulations suggest a simple self-regulatory mechanism for limiting ring stack size where stress from inter-rung rotation builds up until the formation of Interfaces 1 and 3 is no longer favorable beyond a geometrical limit of ∼40°, after which further stacking is impeded. Consistent with this, Interfaces 1 and 3 linking the top two rungs were poorly resolved in Vipp1_C11–C17_ rings, indicating a weak interaction once the limiting geometry is reached. To test the importance of Interface 3 for dome-shaped curvature formation, we mutated two conserved residues, F197K and L200K (Vipp1_F197K/L200K_), within this interface. Although this severely inhibited self-assembly, indicating that Interface 3 was important for both rung formation and ring stability, a minor fraction of Vipp1_F197K/L200K_ still formed rings and filaments. Strikingly, these filaments tended to have a broadly uniform diameter along their length consistent with a loss of inter-rung tilt (Figure S7A). These data suggest that Interface 3 forms a tensile connection between rungs that facilitates the formation of dome-shaped rings.

Video S4. Energy minimized elastic-network model of Vipp1 forms Vipp1-like dome-shaped rings, related to Figure 6C–6E The initial structure is a C11 stack composed of eight identical and energy minimized rungs that contains no inter-rung interactions. Inter-rung interactions were added and the movie traces elastic energy minimisation. The equilibrium structure reached is a dome-shaped structure that balances the inter-rung rotation favored by the inter-rung interactions with the overall distortion in each rung.6

### Vipp1 polymers and membrane remodeling

As well as rings, we observed native Vipp1 and Vipp1Δα6_1–219_ proteins to form multiple types of polymeric assemblies including filaments (Figures S2B, S2D, and S7C). Fourier Bessel analysis suggested that Vipp1 filaments are helical with variable diameters and lattices. For the 14-nm diameter filament, one of the dominant layer lines corresponds to a 30.5 Å repeat, which fits closely to the average axial rise between hairpins within neighboring rungs (Figures 5 and S2D). Given the Vipp1 subunit is >16 nm in length when maximally curved, significant conformational changes or lattice rearrangements may be required to build the 14 nm filament. In this case, Vipp1 may be forming a filament similar to ESCRT-III Vps24, which constitutes a 16-nm diameter double-stranded filament (Huber et al., 2020). Vipp1 stack arrays were also observed morphing from discrete rings into ribbons then filaments, particularly with Vipp1Δα6_1–219_ (Figure S7C). Overall, these micrographs indicate that Vipp1 and Vipp1Δα6_1–219_ can transition between lattices in an extraordinary display of assembly versatility reminiscent of ESCRT-III filaments (Bertin et al., 2020; Chiaruttini et al., 2015; von Filseck et al., 2020; Lata et al., 2008; McCullough et al., 2015, 2018).

**Figure 7:**
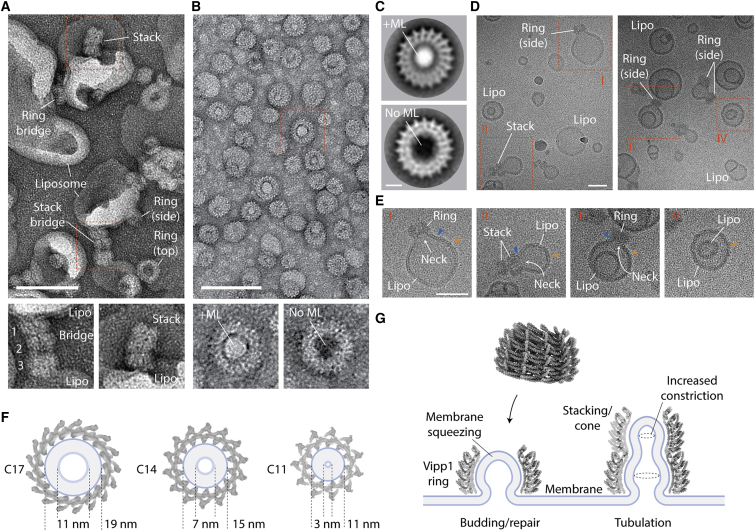
Mechanism of Vipp1 membrane repair and fusion (A) Negative stain electron micrograph showing Vipp1 rings decorating and tethering liposomes together. Individual rings or ring stacks form bridges between liposomes. Scale bar, 100 nm. (B) Vipp1 rings decorate a lipid monolayer (ML). Zoom panels compare rings in the presence or absence of lipid monolayer. Lipid monolayer is drawn into the ring and occludes the lumen (white central density). In the absence of lipid monolayer, rings have an empty lumen (black central density). (C) Class averages of Vipp1 rings in the presence (top) and absence (bottom) of lipid monolayer. Scale bar, 10 nm. (D) Cryo-electron micrographs showing Vipp1 rings decorating liposomes (lipo). Scale bar, 50 nm. (E) Zoom panels of red dotted boxes shown in (D). In contrast to the positively curved liposome surface (orange wedge), negative curvature (blue wedge) is observed as an open neck curling into the base of the bound Vipp1 ring (I–III). An example of an undecorated liposome (IV). Scale bar, 50 nm. (F) A membrane tube comprising 4-nm lipid bilayer is modeled into Vipp1_C17/C14/C11_ rings to show constriction progression. Membrane hemifusion is expected to be achieved within Vipp1_C11_. (G) Schematic showing how Vipp1 may repair damaged or perturbed membrane on a single lipid bilayer. Ring stacks and cones facilitate membrane tubule formation and increasing constriction toward the cone apex. See also Figure S7.

To determine how *N. punctiforme* Vipp1 rings interact with lipids, we first used a spin pelleting assay to demonstrate binding to *E. coli* liposomes (Figure S7E). We then directly observed the interplay between Vipp1 and liposomes by NS EM. Vipp1 rings decorated the liposome surface (Figures 7A and S7F). Side views of Vipp1 bound to liposome edges clearly showed the rings attached to the membrane surface. Tilt and side views, which were only observed associated with a liposome edge, also revealed how dome-shaped rings preferentially attach to the membrane via their wider opening rather than their sides. This suggests that helix α6, which coats the outer surface of the ring, does not mediate membrane binding in this instance (Hennig et al., 2017). Ring stacks were often observed protruding from liposome edges (Figure S7F), together with cones that formed from stacks of rings with decreasing diameters. Rings, stacks, and cones also formed tethers that connected liposomes via their openings (Figures 7A and S7F). In support of a role for the ring lumen in membrane binding, when we added Vipp1 polymers to *E. coli* lipid monolayers (Figures 7B, 7C, and S7G–S7I), rings were only observed bound to the monolayer via their openings. Intriguingly, these rings often had occluded lumens demarcated by white centers (Figure 7C), consistent with a model in which the monolayer was adsorbed onto the inner ring wall forming an encapsulated vesicle-like bud that excludes the negative stain. This model was further supported by side views of Vipp1 rings attached to liposomes in vitreous ice where the positive curvature of the lipid bilayer surface was remodeled to form an open neck of negative curvature as the membrane enters the Vipp1 ring base (Figures 7D and 7E).

## Discussion

Here, we show that Vipp1, PspA, and ESCRT-III constitute an ancient superfamily of related membrane remodeling proteins that are conserved across archaea, bacteria, and eukaryotes. Excitingly, this adds ESCRT-III to the growing list of universal cytoskeletal proteins, like actin and tubulin (van den Ent et al., 2001), which, although once thought to be defining features of eukaryotes, are now known to have their origins in prokaryotes. Further studies are required to determine which features of these ESCRT-III-like polymers (including archaeal PspA, euryarchaeal ESCRT-III-like proteins, as well as bacterial Vipp1/PspA) are generic and which are likely to be domain-, phylum-, or species-specific. For example, it is not clear whether the ATPase PspF (Data S1C; Elderkin et al., 2005) performs a similar role to Vps4 in the control of PspA-dependent membrane remodeling nor whether there is an equivalent ATPase that assists Vipp1 in its membrane remodeling activities.

In addition to homology between PspA/Vipp1 and ESCRT-III at the primary amino acid sequence level (Figure S1), our analyses show that Vipp1 homologs share a similar secondary structure (Figure 1A) and overall fold with ESCRT-III proteins (Figure 4A). Furthermore, when polymerized, Vipp1 shares core assembly features with ESCRT-III polymers like CHMP1B (McCullough et al., 2015; Nguyen et al., 2020), which includes regions of flex that allow both polymers to assume different forms (Figure 5). Notably, Vipp1 contains a similar hairpin motif, elbow, and wrist joints (hinges 2 and 3) as reported for CHMP1B (McCullough et al., 2015; Nguyen et al., 2020). Vipp1 and CHMP1B also share a shoulder joint located at the C terminus of helix α2 (hinge 1). Collectively, these conserved hinges enable the polymers to assume forms that differ widely in curvature and tilt, including a broad variety of complex 3D structures (Bertin et al., 2020; Pfitzner et al., 2020). In addition, both Vipp1 and CHMP1B form polymers through side-by-side packing of the hairpin motif and through helix α5 contacting the hairpin of neighboring subunits *j*+3 or *j*+4, respectively. This helix α5 contact, which forms Interface 1, is conserved in multiple ESCRT-III proteins and represents a defining feature that explains how this superfamily of proteins generates polymers.

Although both Vipp1 and ESCRT-III proteins form helical filaments, here, we have focused our analysis on the architecture of seven 1.6–3.4 MDa Vipp1 rings from the cyanobacterium *N. punctiforme*. The analysis of different ring symmetries, ranging from C11–C17, provides us with a view of the structural flexibility that enables Vipp1, PspA, and ESCRT-III family members to perform their functions. Furthermore, as each ring comprises between 5–7 rungs stacked together to build a dome-shaped architecture, these asymmetric structures provide us with a glimpse of the structural features that enable filament tilt in the context of an ESCRT-III-like polymer. This is important because simulations have shown that filament tilt may facilitate ESCRT-III transition from a planar spiral to a three-dimensional cone to generate force and drive membrane deformation (Harker-Kirschneck et al., 2019; Pfitzner et al., 2020).

Although the physiological role of Vipp1 within the cell is not fully understood, the results of many cell biological studies indicate that this protein family functions to mitigate stress in photosynthetic membranes (Bryan et al., 2014; Zhang and Sakamoto, 2015; Zhang et al., 2012, 2016) as well as in thylakoid membrane biogenesis and repair (Aseeva et al., 2007; Fuhrmann et al., 2009b; Gao and Xu, 2009; Gutu et al., 2018; Kroll et al., 2001; Lo and Theg, 2012; Nordhues et al., 2012; Walter et al., 2015; Westphal et al., 2001; Zhang and Sakamoto, 2015). In addition, some studies have shown that Vipp1 can seal and repair leaky membranes *in vitro* (Junglas et al., 2020; Siebenaller et al., 2020). Similarly, the closely related bacterial PspA is known to mediate inner membrane repair in response to stress (Joly et al., 2010; Kobayashi et al., 2007; McDonald et al., 2015, 2017), whereas the PspA homolog LiaH in *Bacillus subtilis* protects the cell against oxidative stress and cell wall-targeted antibiotics (Wolf et al., 2010), and the homolog Rv2744c in *Mycobacterium tuberculosis* re-locates to the membrane surface of lipid droplets (Datta et al., 2015).

Although the previous reconstructions of both Vipp1 and PspA rings (Hankamer et al., 2004; Saur et al., 2017) do not readily show how such structures might mitigate membrane stress, the Vipp1_C11–C17_ rings presented here suggest a potential mechanism for stabilizing or repairing localized sections of breached or perturbed membrane. They also provide a mechanism for Vipp1-mediated membrane curving and tubulation. When membrane was modeled into the Vipp1_C11–C17_ structures as a 4-nm bilayer, sequential constriction was observed with the membrane lumen reaching ∼3 nm within Vipp1_C11_ (Figure 7F). This is close to the biophysical limit required to induce membrane fusion/fission (Chernomordik et al., 1995; Kozlovsky and Kozlov, 2003). Our biochemical experiments also suggest how Vipp1 rings might help seal membrane breaches or local bilayer perturbations (Figures 7B, 7C, and S7G) by binding to membranes through the lumenal amphipathic α0 helices (Heidrich et al., 2016; McDonald et al., 2015, 2017; Otters et al., 2013). As membrane is drawn into the Vipp1 ring lumen via capillary action (Figure 7D and 7E), since Vipp1 rings are most constricted at their top, the membrane will become increasingly squeezed as it ascends so that breached or perturbed membrane leaflets could converge to a point of stabilization or fusion at the ring apex (Figure 7G). Although it is not clear how ESCRT-III polymers repair membrane (Jimenez et al., 2014; Sønder et al., 2019), the Vipp1 structures suggest a potential mechanism for ESCRT-III polymers acting in this role when bound to the outside of positively curved membranes. Such capillary action could also explain the entry of lipids into the central lumen of pre-formed *Chlamydomonas reinhardtii* Vipp1 helical filaments (Theis et al., 2019).

It is also possible that ensembles of Vipp1 ring-like structures mediate more complex membrane remodeling events similarly to ESCRT-III polymers (Bertin et al., 2020; Nguyen et al., 2020; Pfitzner et al., 2020). This could be achieved by ring stacking, cone, or filament formation, which could support the establishment of membrane tubules (Figure 7G). It is possible that such structures tether and bridge opposed membranes (Figures 7A and S7F) as a precursor to membrane fusion as has been reported *in vitro* (Hennig et al., 2015, 2017; Saur et al., 2017). In any bridging event, it is possible that Vipp1 rings, stacks, or filaments extend from both opposing membranes so that a seam of opposing handedness is created where the polymers meet, as was observed in *C. reinhardtii* Vipp1 protein-lipid tubes (Theis et al., 2019).

In summary, our study shows that PspA, Vipp1, and ESCRT-III constitute an ancient membrane repair/remodeling machine that was likely present in the last universal common ancestor of all cells (LUCA). Building on this evolutionary insight, our structural analysis of Vipp1 reveals the conserved architecture of these proteins, including three hinge regions. Such architecture provides these polymers with the flexibility required to remodel membrane across all domains of life.

### Limitations of the study

The *N. punctiforme* Vipp1_C11–C17_ models (Figure 2) are broadly consistent with concurrently reported *Synechocystis* Vipp1 and PspA structures (Gupta et al., 2021; Junglas et al., 2021, this issue of *Cell*). Future cryo-EM studies using *N. punctiforme* Vipp1 may facilitate higher resolution maps to be resolved with improved accuracy of main chain position and showing side chain detail. Although Vipp1 rings and filaments have been purified from cyanobacteria, algae, and plants, it is still unclear how these polymers relate to cellular Vipp1 forms and membrane remodeling activities. Note similar difficulties arise when trying to reconcile the *in vitro* structures of ESCRT-III polymers associated with membranes (McCullough et al., 2015; Nguyen et al., 2020) with their *in vivo* functions, where in many cases these same proteins are known to function in the reverse topology. Finally, it remains uncertain whether Vipp1 functions as a composite co-polymer with Vipp2 and/or is remodeled via its association with an external ATPase, analogous to the way ESCRT-III polymers work. These outstanding questions represent an exciting frontier for future studies.

## STAR★Methods

### Key resources table

**Table:** 

REAGENT or RESOURCE	SOURCE	IDENTIFIER
**Bacterial and virus strains**		

*E. coli* C43 (DE3)	Lucigen	60345-1
*E. coli* C43 (DE3) *pspA*^*-*^	Chernyatina and Low, 2019; Datsenko and Wanner, 2000	N/A
*E. coli* BL21 (DE3)	Lucigen	60300-1

**Chemicals, peptides, and recombinant proteins**		

Tris- Base	Sigma	CAS 77-86-1
Tris- HCl	Sigma	CAS 1185-53-1
HEPES sodium salt	Sigma	CAS 75277-39-3
NaCl	Sigma	CAS 7647-14-5
KCL	Sigma	CAS 7447-40-7
EDTA	Sigma	CAS 6381-92-6
DTT	Melford	CAS 3483-12-3
IPTG	Melford	CAS 367-93-1
Ampicillin	Melford	CAS 69-52-3
MgCl_2_	Sigma	CAS 7786-30-3
GMPPCP	Sigma	CAS 10470-57-2
LB-Agar	Miller	1102830500
Lysozyme from chicken egg white	Sigma	CAS 12650-88-3
Deoxyribonuclease I from bovine pancreas	Sigma	CAS 9003-98-9
2xYT Broth	Melford	38210000
Dichloro(1,10-phenanthroline)copper(II) (CuP)	Sigma	CAS 14783-09-6
Ethanol absolute	Sigma	CAS 64-17-5
Chloroform	Sigma	CAS 67-66-3
N-ethylmaleimide	Sigma	CAS 128-53-0
1,4-Butanediyl Bismethanethiosulfonate (MTS4)	Santa Cruz Biotechnology	CAS 55-99-2
TEV protease	In-house purification	N/A
Dynamin 1	In-house purification	N/A
D-(+)- Maltose monohydrate	Fluorochem	CAS 6363-53-7
Amylose Resin	NEB	E8021L
*E. coli* total lipid extract	Avanti polar lipids	CAS 1240502-50-4
Sodium dodecyl sulfate (SDS)	Fisher Scientific	CAS 151-21-3
Llithium dodecyl sulfate (LDS) sample buffer (4X)	Invitrogen	B0007
cOmplete, EDTA-free protease inhibitor cocktail tablets	Roche	11873580001
Precision Plus Protein Unstained Protein Standards, Strep-tagged recombinant	Biorad	1610363

**Deposited data**		

Vipp1 C11	This paper	EMDB-11468, PDB 6ZVR
Vipp1 C12	This paper	EMDB-11469, PDB 6ZVS
Vipp1 C13	This paper	EMDB-11470, PDB 6ZVT
Vipp1 C14	This paper	EMDB-11478, PDB 6ZW4
Vipp1 C15	This paper	EMDB-11481, PDB 6ZW5
Vipp1 C16	This paper	EMDB-11482, PDB 6ZW6
Vipp1 C17	This paper	EMDB-11483, PDB 6ZW7
CHMP3	Bajorek et al., 2009	3FRT
CHMP1B	McCullough et al., 2015	3JC1
CHMP1B	Nguyen et al., 2020	6TZ4
PspA	Osadnik et al., 2015	4WHE

**Oligonucleotides**		

P1_F TTCCAGGGCTCCCATATGGGA TTATTCGATCGCATTAAG	Eurofins	N/A
P2_B ATGATGATGGGATCTTTATAGTTG ATCCAATTGCTTGCG	Eurofins	N/A
P2_F ATGCTACCATAAAAGC TTGGTACCACGCGTGC	Eurofins	N/A
P2_R AAGCTTTTATGGTAGCATT TGCGCTTTCAAAGC	Eurofins	N/A
P3_F GCAGGTGCATAAAAGCTT GGTACCACGCGTGC	Eurofins	N/A
P3_R AAGCTTTTATGCACCTGCT AACTCTCCTAGTGC	Eurofins	N/A
P4__F GAGAATTGTGCACGACAAGCTTTAGAGCG	Eurofins	N/A
P4__R TTGTCGTGCACAATTCTCAT CACCTTTTTGTAGGGCG	Eurofins	N/A
P5_F GCAGATTGTGAAACCCAATTTGCCCAGTTGG	Eurofins	N/A
P5_R TTGGGTTTCACAATCTGCAC CTGCTAACTCTCCTAG	Eurofins	N/A
P6_F CAAAAAGCCCAGAAAGAAGGT GGTAGCGATGTTGATGATGAATTA	Eurofins	N/A
P6_R TTCTTTCTGGGCTTTTTGG GTTTCTAAATCTGCACCTGC	Eurofins	N/A

**Recombinant DNA**		

pOPTM/Vipp1	This work	N/A
pOPTM/Vipp1Δα6_1-219_	This work	N/A
pOPTM/Vipp1Δα5/6_1-191_	This work	N/A
pOPTM/Vipp1_L86C_	This work	N/A
pOPTM/Vipp1_L193C_	This work	N/A
pOPTM/Vipp1_L86C/L193C_	This work	N/A
pOPTM/Vipp1_F197K/L200K_	This work	N/A

**Software and algorithms**		

HHMER	Finn et al., 2011	http://hmmer.org/
HHPred	Zimmermann et al., 2018	https://toolkit.tuebingen.mpg.de/tools/hhpred
Phyre2	Kelley et al., 2015	http://www.sbg.bio.ic.ac.uk/phyre2/html/page.cgi?id=index
Psipred	Buchan and Jones, 2019	http://bioinf.cs.ucl.ac.uk/psipred/
Gremlin	Anishchenko et al., 2017; Ovchinnikov et al., 2014	https://gremlin2.bakerlab.org/exceptions.php
AnnoTree	Mendler et al., 2019	http://annotree.uwaterloo.ca/
mafft 7.3.1	Katoh and Standley, 2013	https://sbgrid.org/software/titles/mafft
trimAl 1.3	Capella-Gutiérrez et al., 2009	http://trimal.cgenomics.org/
IQ-Tree 1.6.10	Nguyen et al., 2015	http://www.iqtree.org/release/v1.6.10
Relion 3.1	Scheres, 2012	https://www3.mrc-lmb.cam.ac.uk/relion/index.php?title=Main_Page
I-Tasser	Zhang, 2008	https://zhanglab.dcmb.med.umich.edu/I-TASSER/
COOT	Emsley et al., 2010	https://www2.mrc-lmb.cam.ac.uk/personal/pemsley/coot/
Rosetta	Wang et al., 2016	https://www.rosettacommons.org/software
Chimera	Pettersen et al., 2004	https://www.cgl.ucsf.edu/chimera/
ISOLDE	Croll, 2018	https://isolde.cimr.cam.ac.uk/
PHENIX	Adams et al., 2010	https://phenix-online.org/
Molprobity	Chen et al., 2010	http://molprobity.biochem.duke.edu/
Imagic	van Heel et al., 1996	https://www.imagescience.de/imagic.html
Ximdisp	Smith, 1999	https://www2.mrc-lmb.cam.ac.uk/research/locally-developed-software/image-processing-software/
ImageJ	Schneider et al., 2012	https://imagej.nih.gov/ij/

### Resource availability

#### Lead contact

Further information and requests for resources and reagents should be directed to and will be fulfilled by the lead contact, Harry Low (h.low@imperial.ac.uk).

#### Materials availability

All unique/stable reagents generated in this study are available from the Lead Contact without restriction.

#### Data and code availability

3D cryo-EM density maps produced in this study have been deposited in the Electron Microscopy Data Bank with accession code EMD-11468, EMD-11469, EMD-11470, EMD-11478, EMD-11481, EMD-11482 and EMD-11483 for Vipp1_C11-C17_, respectively. Atomic coordinates have been deposited in the Protein Data Bank (PDB) under accession code 6ZVR, 6ZVS, 6ZVT, 6ZW4, 6ZW5, 6ZW6 and 6ZW7 for Vipp1_C11-C17_, respectively.

### Experimental model and subject details

All Vipp1 proteins were overexpressed and purified from *E. coli* C43 (DE3) electro-competent cells (Lucigen) modified to incorporate a *pspA* gene knockout. Dynamin 1 was overexpressed and purified from *E. coli* BL21 (DE3) electro-competent cells (Lucigen). Further details including culture conditions are outlined below in Method details.

### Method details

#### ESCRT-III and PspA/Vipp1 evolutionary analyses

To search for ESCRT-III relatives, we used sensitive protein sequence searches (HHMER [Finn et al., 2011], HHPred [Zimmermann et al., 2018] and Pfam [El-Gebali et al., 2019]) based on Hidden Markov Models (HMMs). Several individual eukaryotic ESCRT-III proteins, multiple sequence alignments and HMM profiles were used as queries in these searches. These analyses consistently identified PspA/Vipp1 proteins as the only ESCRT-III homologs in sequence, domain and structural databases (Uniprot [UniProt Consortium, 2019], GenBank [Sayers et al., 2019], Pfam [El-Gebali et al., 2019] and PDB [Burley et al., 2019]). This observation was corroborated by the fact that these protein families share a common Pfam clan which only includes PspA and ESCRT-III (Pfam CL0235). Secondary structure predictions were performed using HHPred (Zimmermann et al., 2018), Phyre2 (Kelley et al., 2015) and Psipred (Buchan and Jones, 2019) software. To obtain a statistical model of the PspA/Vipp1 family that captures patterns of residue co-evolution, Gremlin (Anishchenko et al., 2017; Ovchinnikov et al., 2014) was used. For this analysis, a total of 2844 homologous proteins were obtained using *N. punctiforme* Vipp1 as a query, four iterations of JackHHMER searches, E-value ≤ 1e-10, using a coverage filter of 50% and gap removal of 75%.

#### Phylogenetic analyses

The phylogenetic distribution of the PspA/Vipp1 and ESCRT-III families was generated using AnnoTree (Mendler et al., 2019) searches in Pfam (El-Gebali et al., 2019) and an E-value ≤ 1e-05. Over 27000 bacterial and 1500 archaeal genomes were analyzed. For the generation of a phylogenetic tree of the Vipp1-ESCRT-III superfamily, homologs of these proteins were retrieved from Uniprot (UniProt Consortium, 2019) by HMMER (Finn et al., 2011) searching and from InterPro (Mitchell et al., 2019) database, followed by manual inspection. Eukaryotic, archaeal and bacterial PspA and ESCRT-III proteins were selected to achieve a broad distribution of homologs across the tree of life. In total, 264 PspA and 332 ESCRT-III sequences were selected, aligned using the l-INS-I mode in mafft 7.3.1 (Katoh and Standley, 2013), and poorly-aligned regions were identified and removed using the “gappyout” mode in trimAl 1.3 (Capella-Gutiérrez et al., 2009). The phylogeny was inferred in IQ-Tree 1.6.10 (Nguyen et al., 2015) under the LG+C30+G+F model, which was the best-fitting model according to the BIC criterion. This model accounts for differences in exchange rates among amino acids (LG), different site compositions (C30+F), and models across-site rate variation using a discretized Gamma distribution with 4 rate categories. Branch supports are ultrafast (UFBoot2 [Hoang et al., 2018]) bootstraps. The same steps above were used for the generation of the phylogenetic tree of ATPases, with the only differences being that 194 ATPase sequences were selected and the best-fit model chosen by BIC was LG+R8, i.e., with across-site rate variation modeled using a mixture of eight rates that were not constrained to be drawn from a Gamma distribution. Site-specific evolutionary rates, measured in units of expected number of substitutions per site, were inferred using the empirical Bayes method in IQ-TREE (–rate) from subfamily-specific (ESCRT-III and PspA/Vipp1) sequence alignments. The analysis of the number of copies per genome of PspA/Vipp1 and ESCRT-III/Snf7 genes were performed inspecting a variable number of genomes per taxonomic group, using HHMER searches and the following proteins as queries (species and protein name, Uniprot code): PspA/Vipp1 - 133 bacterial genomes (*N. punctiforme* Vipp1, B2J6D9; this analysis excludes cyanobacterial genes), 421 archaea (*Haloferax volcanii* PspA, D4GUW2), 180 eukaryotes containing chloroplasts (*C. reinhardtii* Vipp1, A8JC26), 502 cyanobacteria (*N. punctiforme* Vipp1, B2J6D9); ESCRT-III/Snf7 - 40 Euryarchaeota (*Halomicrobium mukohataei* ESCRT-III-like, C7P4W4), 13 Asgard archaea (*Candidatus Odinarchaeota* archaeon LCB_4 Vps2/24/46 and Vps20/32/60 homologs, A0A1Q9N7Y8 and A0A1Q9N7Y5) and 165 TACK archaea (*Sulfolobus acidocaldarius* CdvB, Q4J924).

#### Vipp1 cloning, expression and purification

Plasmid mutagenesis and all clones were generated using the Gibson isothermal DNA assembly protocol (Gibson et al., 2009). Plasmids and primers used in this study are listed in Table S2. The coding sequence for *N. punctiforme vipp1* (Uniprot code B2J6D9) was cloned into pOPTM (a pET derivative) to yield an N-terminal MBP fusion with a TEV cleavage site in the linker. An N-terminal hexahistidine tag was included on the MBP moiety. For the purification of both native and mutant Vipp1, clones were co-transformed into *E. coli* C43 (DE3) electro-competent cells (Lucigen) modified to incorporate a *pspA* gene knockout using a Lambda Red recombinase strategy (Chernyatina and Low, 2019; Datsenko and Wanner, 2000). Cells were grown on selective LB-agarose plates with ampicillin (100 μg/ml). 2xYT media was inoculated and cells grown at 37°C until induction at OD_600_ = 0.8 with 1 mM isopropyl β-D-1- thiogalactopyranoside (IPTG). Cells were grown for ∼15 h at 18°C and shaken at 220 rpm. All the further steps were carried out at 4°C unless otherwise specified. Purification of Vipp1_L86C_, Vipp1_L193C_ and Vipp1_L86C/L193C_ were performed in the presence of 2 mM dithiothreitol (DTT). Pellets were re-suspended in ice-cold buffer 50 mM Tris- HCl pH 7.5, 300 mM NaCl, treated with 2 mM MgCl_2_, 0.1 mg/ml DNase I, 0.5 mg/ml lysozyme and sonicated on ice. The lysate was clarified by centrifugation at 16,000 × g for 20 min. The supernatant was incubated with gentle shaking for 1 h with 10 mL of amylose resin (NEB) pre-equilibrated in 50 mM Tris- HCl pH 7.5, 300 mM NaCl (wash buffer). The resin was washed with 100 mL and purified MBP-Vipp1 eluted with wash buffer supplemented with 15 mM maltose. The sample was incubated for 24 h at room temperature with TEV and then dialysed (12-14 kDa MW cut-off) overnight in 25 mM Tris- HCl pH 8.4, 40 mM NaCl. The sample was concentrated and injected onto a sephacryl 16/60 S500 gel filtration column equilibrated in 25 mM Tris- HCl pH 8.4, 50 mM NaCl. A typical elution profile for Vipp1 consisted of three peaks containing 1) Vipp1 superstructures such as filaments eluting at ∼40 mL within the column size exclusion limit or void volume, 2) Vipp1 rings eluting at ∼65 mL, 3) non-polymerized low molecular weight Vipp1 species consistent with monomer or dimers, MBP and TEV eluting at ∼100 mL. Fractions from peak 1 and 2 were pooled and concentrated up to 1 mg/mL. Where necessary, the sample was gel filtrated a second time to reduce residual MBP or TEV contamination. LC-MS/MS confirmed the identity of the Vipp1 band identified by SDS-PAGE. Note that native Vipp1 migrates at ∼38 kDa and not at its expected molecular weight of 28.7 kDa. As Vipp1Δα5/6_1-191_ gel filtrates only in peak 3, the removal of MBP and TEV was necessary for a clean SDS-PAGE analysis. An additional affinity chromatography step was therefore included directly before gel filtration using 2 × 5 mL HisTraps (GE Healthcare). As both the MBP and TEV proteins incorporate a hexahistidine tag, the flow through containing Vipp1Δα5/6_1-191_ was collected for subsequent steps.

#### Dynamin cloning, expression and purification

The rat *dynamin 1* gene (Uniprot code P21575) with the PRD domain truncated was cloned into pOPTM vector yielding an N-terminal MBP fusion with a TEV cleavage site in the linker. A C-terminal hexahistidine tag was included on Dynamin 1. *E. coli* thioredoxin was inserted at the Dynamin 1 N terminus through Gibson assembly. The role of the thioredoxin, which was not cleaved off, was to stop the large-scale clumping of Dynamin 1 filaments in solution and to facilitate a broad distribution of filaments on the EM grid. Transformed *E. coli* BL21 (DE3) cells were grown on selective LB-agarose plates with ampicillin (100 μg/ml). 2xYT media was inoculated and cells grown at 37°C until induction at OD_600_ = 0.6 with 1 mM isopropyl β-D-1- thiogalactopyranoside (IPTG). Cells were grown for ∼15 h at 19°C and shaken at 220 rpm. All the further steps were carried out at 4°C unless otherwise specified. For purification, pellets were re-suspended in 50 mM Tris- HCl pH 8.0, 500 mM NaCl, 2 mM DTT, 2 mM EDTA and sonicated on ice. The lysate was clarified by centrifugation in a Ti45 rotor (Beckman Coulter) at 98,000 × g at 4 °C for 45 min and the supernatant loaded onto a self-packed column with ∼10 ml of amylose resin (NEB) pre-equilibrated in 50 mM Tris- HCl pH 9.0, 500 mM NaCl, 1 mM DTT, 1 mM EDTA and 20% glycerol (wash buffer). The resin was washed with 200 mL of wash buffer and the MBP-Dynamin 1 eluted with wash buffer supplemented with 15 mM maltose. The sample was incubated overnight at room temperature with TEV and the products separated by gel filtration using a HiPrep 26/60 Sephacryl S300 column in buffer 20 mM Tris- HCl pH 9.0, 1mM EDTA and 1 mM DTT. Fractions of Dynamin 1 were concentrated to ∼10 mg/ml, flash frozen in liquid nitrogen and stored at −80 °C.

#### Cryo-EM preparation and data collection

Under cryogenic conditions *N. punctiforme* Vipp1 exhibits significant preferred orientation so that essential side views for determining the structure were absent. To solve this issue, preformed Dynamin 1 filaments with 37 nm diameter were mixed with Vipp1 prior to vitrification. Rat Dynamin 1 filaments appear equivalent to human Dynamin 1 filaments in the super constricted state (Sundborger et al., 2014). The Dynamin 1 filaments formed a network on the grid that helped to maintain ice thickness around Vipp1 rings so that positioning at the air-water interface was reduced and side views captured. 37.5 μM Dynamin 1 was incubated with 20 mM HEPES-NaOH pH 7.2, 50 mM NaCl, 1 mM DTT, 2mM GMPPCP and 5 mM MgCl_2_ at room temperature for 2 hours to form filaments. 35 μM Vipp1 was then mixed with the pre-formed dynamin filaments. 4 μL of the mixture was incubated for 30 s on glow discharged holey R2/2 Quantifoil grids before vitrification in liquid ethane using a Vitrobot Mark IV (FEI). Data were collected at 300 kV on a Titan Krios (M02 beamline at eBIC Diamond, UK) equipped with a Gatan Quantum K2 Summit detector. 3206 movies were acquired at a magnification of 35,971 yielding 1.39 Å/pixel using EPU software. Defocus was between −1.25 and −3.0. Movies were dose-weighted over 40 frames with 10 s exposures. Total dose was 50 e/Å^2^.

#### Cryo-EM image processing

Individual movie frames were aligned with MotionCor2 (Zheng et al., 2017) and the contrast transfer function estimated using CTFFIND4 (Rohou and Grigorieff, 2015). All subsequent processing was carried out using Relion 3.0 (Scheres, 2012). Particles were picked manually to generate initial 2D class averages that were subsequently used for reference-based auto-picking. Extracted particles were subjected to five rounds of 2D classification resulting in a cleaned stack of 109,715 particles. To generate the initial 3D model, a subset of 2D classes comprising C14 symmetry top views and side views with diameter range from 28 nm to 32 nm were selected (24,355 particles). 3D classification was carried out in C1 using a featureless hollowed cylinder as the initial reference. One class containing 9,991 particles yielded a ring with distinct C14 symmetry, which was chosen for high-resolution reconstruction first. These particles were then 3D autorefined with C14 symmetry applied reaching 8.5 Å resolution. The resulting map (Intermediate map 1) showed clear secondary structure features and was used as the new C14 reference volume for a second round of processing. In round two, side views only of 2D class averages were selected and 3D classified iteratively in C1 using Intermediate map 1 as a reference volume. In this way 15,767 side views with C14 symmetry were isolated. These side views were combined with 3,663 C14 top view particles obtained during 2D classification. A 3D autorefinement was undertaken with C14 symmetry applied to yield a reconstruction at 7.0 Å resolution (Intermediate map 2). Individual particles were then corrected for beam-induced motion for a third round of processing. One round of 3D classification was undertaken in C1 using Intermediate map 2 as the reference volume. A final stack of 17,114 particles was then used for 3D autorefinement with C14 symmetry applied reaching 6.8 Å resolution. Post-processing yielded 6.5 Å resolution with an auto-estimated B-factor (Rosenthal and Henderson, 2003) of −291.9 Å^2^ applied to sharpen the final 3D map. Resolutions reported are based on gold standard Fourier shell correlations (FSC) = 0.143. Once the Vipp1_C14_ structure was built and targeted masks of asymmetric units or individual rungs could readily be generated, multiple subtraction based local refinements including symmetry expansion strategies were attempted but no improvement in resolution was observed. A similar strategy as implemented for Vipp1_C14_ was carried out to generate all other ring symmetries including Vipp1_C11-C13_ and Vipp1_C15-C17_. A B-factor of −320 Å^2^ was applied to these maps. The hand of the electron density maps was unambiguously determined by fitting the PspA crystal structure (PDB: 4WHE), which has a distinct axial twist and asymmetry. Statistics for data collection and 3D refinement for all maps are included in Table S1.

#### Model building and refinement

Rung 3 of the Vipp1_C14_ structure was built first. A secondary structure prediction was obtained using Psipred (Buchan and Jones, 2019). A partial Vipp1 homology model based on PspA (PDB: 4WHE) was generated using I-Tasser (Zhang, 2008). The model was trimmed to include amino acids 24-142, which represents the hairpin motif. Importantly for obtaining an accurate sequence register in the Vipp1 structure, Vipp1 aligns robustly with PspA in this region with 32.5% sequence identity, 59% similarity and crucially 0% gaps. The hairpin readily fitted into the Vipp1_C14_ map requiring only minor adjustments. Overall, the hairpin from Vipp1_C14_ rung number 3 (PDB: 6ZW4) and PspA hairpin motifs have a Cα RMSD = 2.2 Å (Figure S4E). The hairpin homology model provided an important anchor for subsequently building the N-terminal helix α0 and C-terminal helices α4 and α5. The resolution for the bulk of Vipp1_C14_ within rungs 3 and 4 was ∼5 Å so that the main chain could be easily traced and helices α0-α5 clearly assigned and built using COOT (Emsley et al., 2010). Significant attention was paid to regions of high sequence conservation as a guide for sequence register within predicted interfaces. Similarly, our co-evolutionary contact maps (Figure 1B) were used to confirm interfaces and expected sequence register. Ultimately, the accuracy of the sequence register was experimentally assessed by the introduction of a cysteine pair within Interface 3 and tracking cross-links (Figure 3A). Rosetta (Wang et al., 2016) was used to improve the geometry. The subunit from rung 3 was copied and rigid body fitted into all other rungs within the asymmetric unit using Chimera Fit in map command (Pettersen et al., 2004). COOT and ISOLDE (Croll, 2018) were used to adjust for rung specific conformational changes. Using PHENIX (Adams et al., 2010), non-crystallographic symmetry (NCS) was applied to each asymmetric unit to generate a complete Vipp1_C14_ 84 chain model. This model was truncated to main chain and rigid body and B factor refined in PHENIX. For all other ring symmetries, the Vipp1_C14_ asymmetric model was fitted using Chimera Fit in map command. COOT and ISOLDE (Croll, 2018) were used to adjust for conformational changes specific to ring symmetry. For each ring, NCS was applied to generate complete ring models. All subsequent steps were as for Vipp1_C14_. The final models were assessed using Molprobity and statistics outlined in Table S1 (Chen et al., 2010). The correlation between map and model (CC_mask_) as generated by the phenix.map_model_cc command was C11- 0.8, C12- 0.82, C13- 0.77, C14- 0.84, C15- 0.8, C16- 0.67, C17- 0.58.

#### Helical filament processing

For Vipp1_F197K/L200K_ and Vipp1Δα6_1-219_ filaments (Figures S7A–S7D), a dataset comprising 100 and 121 micrographs, respectively, were collected manually on a Tecnai F20 microscope equipped with Falcon II direct electron detector. Single frames were collected with 1 s exposure, total dose ∼15 e/A^2^, a magnification of 68,293 and a pixel size = 2.05 Å. For Vipp1_F197K/L200K_, using the helical processing module in Relion 3.1, 20,745 segments were extracted and binned to a final pixel size = 6.15 Å. After 2D classification, an aligned stack from a selected class average was created containing 780 segments. The stack was summed using Imagic (van Heel et al., 1996), padded in Ximdisp (Smith, 1999) and ImageJ used for Fourier Transform analysis (Schneider et al., 2012). For Vipp1Δα6_1-219_, 20,745 segments were extracted and binned to a final pixel size = 6.15 Å. 226 segments were used for the aligned stack and subsequent processing. For native Vipp1 14 nm and 24 nm filaments (Figures S2D and S2E), 14,292 segments were extracted from the Vipp1 ring dataset and binned to a final pixel size = 4.17 Å. 9820 and 429 segments contributed to the final class average for the 14 nm and 24 nm filaments, respectively.

#### Elastic network model

To understand the relationship between inter-rung stacking and the creation of dome-shaped curvature in Vipp1 rings, molecular modeling was used focused on the smallest ring system- Vipp1_C11_ for simplicity. Each residue was represented by a single bead at the position of the Cα atom. The potential energy of the structure was defined by an elastic network model (ENM), meaning that interactions between residues nearby in the experimental structure were restrained by harmonic springs. Nearby in the contact map was defined by Cα distances within 10 Å. All springs were given the same stiffness.

The elastic network for each rung was identical, both internally (intra-rung) and in the interactions made with the rungs above it and below it (inter-rung). While the experimental Vipp1_C11_ structure enforced each monomer to be identical within a rung, the monomers between rungs showed small differences. To create the intra-rung network, the contact map for all monomers in rungs with a complete structure (11^∗^4 monomers in Vipp1_C11_ rungs 2-5) was compared and a spring was created for each contact provided it existed in > 50% of the monomers. As the conformational changes in the monomers between different rungs are small, only a few contacts were removed in this process. Those removed were localized to the regions showing the largest shifts between rungs, namely Hinges 2 and 3 within the Vipp1 monomer (Figure 5B). Removing these outlier contacts allows the network to better model the inherent flexibility of the Vipp1 monomer. Note that the intra-rung contacts included contacts between monomers within the same rung. The natural length of each spring was defined as the average of its contact distances over the monomers. In this way, an ENM for an average rung was created. The average rung best matched rung 3, with a Cα RMSD of 0.5 Å. The inter-ring contact map was defined by the interactions between Vipp1_C11_ rungs three and four (Figure 2B) as rung 5 at the bottom is incomplete.

We then computed the equilibrium structure for different stack sizes by minimizing the elastic energy. This minimization was performed by molecular dynamics (MD) at a low temperature followed by a steepest decent minimization. SMOG2 (Noel et al., 2016) with the template “ENM” was used to create topology files for the MD software GROMACS (Abraham et al., 2015) using the Vipp1_C11_ PDB structure as input (PDB: 6ZVR). These topology files were processed as described in the previous paragraph. Initial structures for minimization were created in VMD by manually copying rung three and translating it N times, where N is the desired number of rungs.

The equilibrium structures resulted from balancing the competing effects of 1) the inter-rung interactions driving curvature and 2) the geometrical constraints of doming. While in principle some of the strain could be alleviated by breaking links, this is not allowed in the ENM. This constraint is not present in the experimental system, which may explain why map densities for parts of the upper and lower rungs are less resolved. The rotations between rungs in the equilibrium structures were analyzed by measuring the angle formed by helix α5 with the ring central axis (Figures 6B and 6C). The axis is defined by the z axis in the experimental structure. The line along the direction of helix α5 is defined by two points taken as the centers of mass of residues 194-202 and residues 211-219.

#### Vipp1 cysteines crosslinking assay

DTT was removed from 1 mg/mL Vipp1_L86C_, Vipp1_L193C_ or Vipp1_L86C/L193C_ using PD MiniTrap G-25 columns (GE Healthcare) equilibrated in 25 mM Tris- HCl pH 8.4, 50 mM NaCl. Samples were diluted to 5 μM and incubated with either 10 mM DTT, ortho-Cu(II)1,10-phenanthroline (CuP, stock 10 mM in 20% ethanol) or 5 μM 1,4-Butanediylbismethanethiosulfonate (MTS-4, stock 50 mM in chloroform) for 1 h at room temperature. Vipp1_L86C/L193C_ cross-linked samples were rescued with 10 mM DTT for 1 h at room temperature. Non-reacted cysteines were blocked by the addition of 10 mM N-ethylmaleimide (NEM, stock 0.5 M in 100% ethanol). Samples were evaluated by SDS-PAGE.

#### Liposome preparation

Liposomes were prepared using *E. coli* total lipid extract (Avanti polar lipids, US). Lipid extract was dissolved in chloroform at 25 mg/mL in a glass vial (Thermo Fisher Scientific). Chloroform was evaporated and the lipid dried for 1 h in a vacuum desiccator. The residual lipid film coating the bottom of the vial was hydrated using liposome reaction buffer (20 mM HEPES, pH 8.0 and 80 mM KCl) at a concentration of 6 mg/ml. The lipid was resuspended by vortexing and gentle sonication with a needle tip for 2 min on ice. The suspension was extruded through polycarbonate membranes with 1 or 0.2 μm pore size using a mini-extruder (Avanti Polar Lipids) to create large or small unilamellar vesicles (LUV/SUV). LUV and SUV were stored at 4°C for subsequent use.

#### Vipp1 liposome binding assays and EM

Liposome binding assays were performed by incubating freshly prepared 2 mM SUV with and without purified 5 μM Vipp1 for 2 h at room temperature in liposome reaction buffer. For negative stain EM, 5 μL of each sample was loaded onto glow-discharged 200-mesh carbon coated copper grids and stained with 2% uranyl acetate (UA). Images were acquired using a FEI Tecnai Spirit microscope equipped with a 2 K Eagle camera. For cryo-EM, 4 μL of sample was loaded onto plasma-cleaned ultrathin lacey carbon supported grids (Agar Scientific) and incubated for 90 s before vitrification in liquid ethane using a Vitrobot Mark IV (FEI). Cryo-EM images were collected manually on a Tecnai F20 microscope equipped with Falcon II direct electron detector. Single frames were collected with 1 s exposure, total dose ∼15e/A^2^, a magnification of 109,375 and a pixel size = 1.28 Å.

#### Spin Assay

To detect Vipp1 membrane binding a spin assay was used. 10.5 μM Vipp1 was ultra-centrifuged at 50,000 x g at 20°C for 15 min using a TLA100 rotor to remove any initial aggregation. The supernatant from this first spin was collected and incubated with and without 2 mg/ml LUV for 1 h at room temperature. Samples were subjected to a second spin at 30,000 x g at 20°C for 30 min. The pellet (P) and the supernatant (S) were harvested, made up to equal volumes in LDS sample buffer and analyzed by SDS-PAGE.

#### Vipp1 monolayer assays

Lipid monolayers were prepared using *E. coli* total lipid extract (Avanti Polar Lipids). A custom-made Teflon block containing 4 mm x 4 mm diameter wells were filled with 50 μL of assay buffer (20 mM Tris-HCl, pH 8 and NaCl 50 mM). A 5 μL drop of 0.1 mg/ml lipid dissolved in chloroform was applied to the top of the buffer solution and the chloroform left to evaporate for 1 h. A non-glow discharged carbon coated copper grid was gently placed on top of the lipid layer with the carbon side faced toward the lipid layer. Subsequently, 14 μM Vipp1 was injected into the well using a side port. The control wells containing either monolayer (no protein) or protein only (a drop of chloroform but no lipid) were set up in parallel. Samples were incubated for 2 h before grids were recovered and immediately stained with 2% UA and imaged using a FEI Tecnai Spirit microscope equipped with a 2 K Eagle camera. Images were taken at a magnification of 40,059 and 3.37 Å pixel size. 263 and 156 images were collected of Vipp1 with and without lipid monolayer, respectively, as described above. Gctf1.06 (Zhang, 2016) was used for estimating the contrast transfer function. Processing steps including particle picking and extraction, and 2D classification were carried out using Relion 3.1 (Scheres, 2012). The final class averages were generated from stacks comprising 11,972 and 2,165 particles for Vipp1 with and without lipid monolayer.

### Quantification and statistical analysis

In Figures S3C–S3I and Table S1, the resolution of the Vipp1_C11-C17_ cryo-EM maps was derived from the FSC = 0.143 criterion (Chen et al., 2013).

## Supplemental information

Document S1. Tables S1 and S2

Data S1. Phylogenetic dendrograms, related to Figure 1
