# Supplementary material for: Bacterial Vipp1 and PspA are members of the ancient ESCRT-III membrane-remodeling superfamily
Source: Cell. 2021 Jul 8;184(14):3660–3673.e18. doi: 10.1016/j.cell.2021.05.041 (PMC8281802; doi:10.1016/j.cell.2021.05.041)
Supplement: Document S1. Tables S1 and S2 [file mmc1.pdf]

Cell, Volume *184*

Supplemental information

Bacterial Vipp1 and PspA are members of the ancient ESCRT-III membrane-remodeling superfamily

Jiwei Liu, Matteo Tassinari, Diorge P. Souza, Souvik Naskar, Jeffrey K. Noel, Olga Bohuszewicz, Martin Buck, Tom A. Williams, Buzz Baum, and Harry H. Low

Bacterial Vipp1 and PspA are members of the ancient ESCRT-III membrane-remodelling superfamily

Table S1, Related to Figure 2. Cryo-EM data collection, refinement and validation statistics.

	Vipp1 C11 (EMDB-11468) (PDB 6ZVR)	Vipp1 C12 (EMDB-11469) (PDB 6ZVS)	Vipp1 C13 (EMDB-11470) (PDB 6ZVT)	Vipp1 C14 (EMDB-11478) (PDB 6ZW4)	Vipp1 C15 (EMDB-11481) (PDB 6ZW5)	Vipp1 C16 (EMDB-11482) (PDB 6ZW6)	Vipp1 C17 (EMDB-11483) (PDB 6ZW7)
**Data collection and processing**							
Magnification	35971	35971	35971	35971	35971	35971	35971
Voltage (kV)	300	300	300	300	300	300	300
Electron exposure (e–/Å^2)	50	50	50	50	50	50	50
Defocus range (μm)	1.25-3	1.25-3	1.25-3	1.25-3	1.25-3	1.25-3	1.25-3
Pixel size (Å)	1.39	1.39	1.39	1.39	1.39	1.39	1.39
Symmetry imposed	C11	C12	C13	C14	C15	C16	C17
Final particle number	3,912	7,433	6,920	17,114	18,217	7,017	4,055
Map resolution (Å)	8.2	7.2	7.0	6.5	7.0	7.4	9.7
FSC threshold	0.143	0.143	0.143	0.143	0.143	0.143	0.143
Map resolution (Å)	8.2	7.2	7.0	6.5	7.0	7.4	9.7
**Refinement**							
Homology model template (PDB code)				4WHE			
Map sharpening *B* factor (Å^2)	-320	-320	-320	-291.9	-320	-320	-320
Model composition							
Non-hydrogen atoms	56364	69420	75205	80990	86775	109936	116807
Protein residues	11363	13992	15158	16324	17490	22160	23545
Ligands							
B factors (Å^2)							
Protein	431.62	255.97	323.3	344.77	468.36	635.30	851.99
Ligand							
R.m.s. deviations							
Bond lengths (Å)	0.005	0.005	0.005	0.006	0.005	0.005	0.005
Bond angles (°)	1.119	1.121	1.119	1.253	1.114	1.124	1.125
Validation							
MolProbity score	0.81	0.84	0.92	0.8	1.02	0.95	1.28
Clashscore	1.05	1.24	1.66	1.05	2.43	1.87	5.23
Poor rotamers (%)	0	0	0	0	0	0	0
Ramachandran plot							
Favored (%)	99.61	99.65	99.13	99.48	99.74	99.71	99.78
Allowed (%)	0.39	0.26	0.87	0.52	0.26	0.29	0.22
Disallowed (%)	0.00	0.00	0.00	0.00	0.00	0.00	0.00

Table S2, Related to STAR Methods section. Primers and plasmids

Primer	Sequence	Construct
P1_F	TTCCAGGGCTCCCATATGGGATTATTCGATCGCATTAAG	pOPTM/Vipp1
P1_B	ATGATGATGGGATCTTTATAGTTGATCCAATTGCTTGCG	
P2_F	ATGCTACCATAAAAGCTTGGTACCACGCGTGC	pOPTM/Vipp1Δα6$_{1\text{-}219}$
P2_R	AAGCTTTTATGGTAGCATTTGCGCTTTCAAAGC	
P3_F	GCAGGTGCATAAAAGCTTGGTACCACGCGTGC	pOPTM/Vipp1Δα5/6$_{1\text{-}191}$
P3_R	AAGCTTTTATGCACCTGCTAACTCTCCTAGTGC	
P4_F	GAGAATTGTGCACGACAAGCTTTAGAGCG	pOPTM/Vipp1$_{L86C}$
P4_R	TTGTCGTGCACAATTCTCATCACCTTTTTGTAGGGCG	
P5_F	GCAGATTGTGAAACCCAATTTGCCCAGTTGG	pOPTM/Vipp1$_{L193C}$
P5_R	TTGGGTTTCACAATCTGCACCTGCTAACTCTCCTAG	
P6_F	CAAAAAGCCCAGAAAGAAGGTGGTAGCGATGTTGATGATGAATTA	pOPTM/IM30$_{F197K/L200K}$
P6_R	TTCTTTCTGGGCTTTTTGGGTTTCTAAATCTGCACCTGC	

Introduced mutations in blue

P4 and P5 were also used to generate pOPTM/Vipp1$_{L86C/L193C}$
