# Supplementary material for: Bacterial Vipp1 and PspA are members of the ancient ESCRT-III membrane-remodeling superfamily
Source: Cell. 2021 Jul 8;184(14):3660–3673.e18. doi: 10.1016/j.cell.2021.05.041 (PMC8281802; doi:10.1016/j.cell.2021.05.041)
Supplement: Data S1. Phylogenetic dendrograms, related to Figure 1 [file mmc2.pdf]

Bacterial Vipp1 and PspA are members of the ancient ESCRT-III membrane-remodelling superfamily

Data S1. Phylogenetic dendrograms, Related to Figure 1.

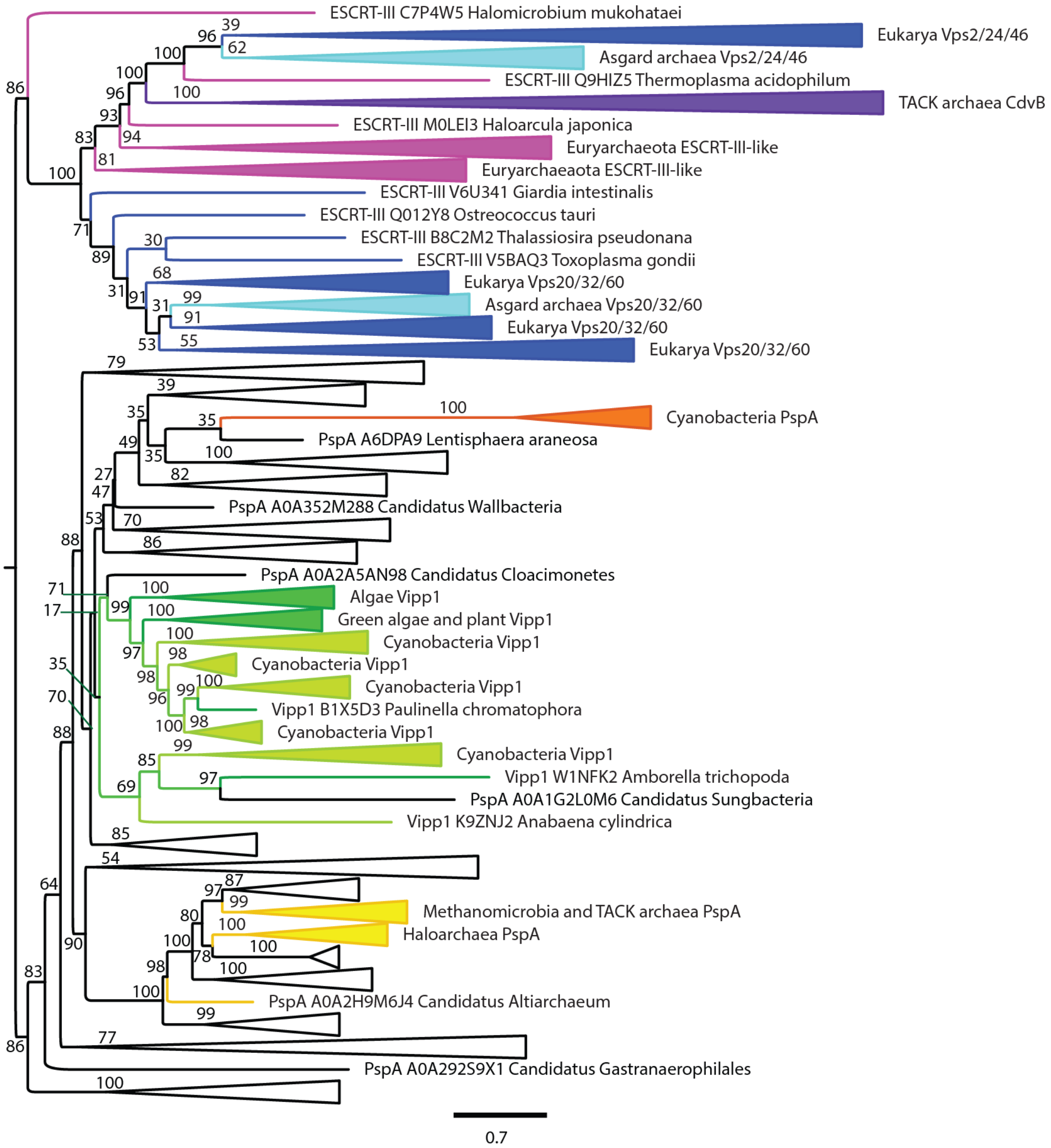

Data S1A. Evolutionary relationship of ESCRT-III/Snf7/CdvB and PspA/Vipp1 protein families. This is the same tree shown in Figure 1D and using equivalent colour scheme, with the exception here that unlabelled white collapsed branches contain bacterial PspA homologues. Numbers indicate the relative support from 100 bootstrap replicates. Scale bar represents expected substitutions per site.

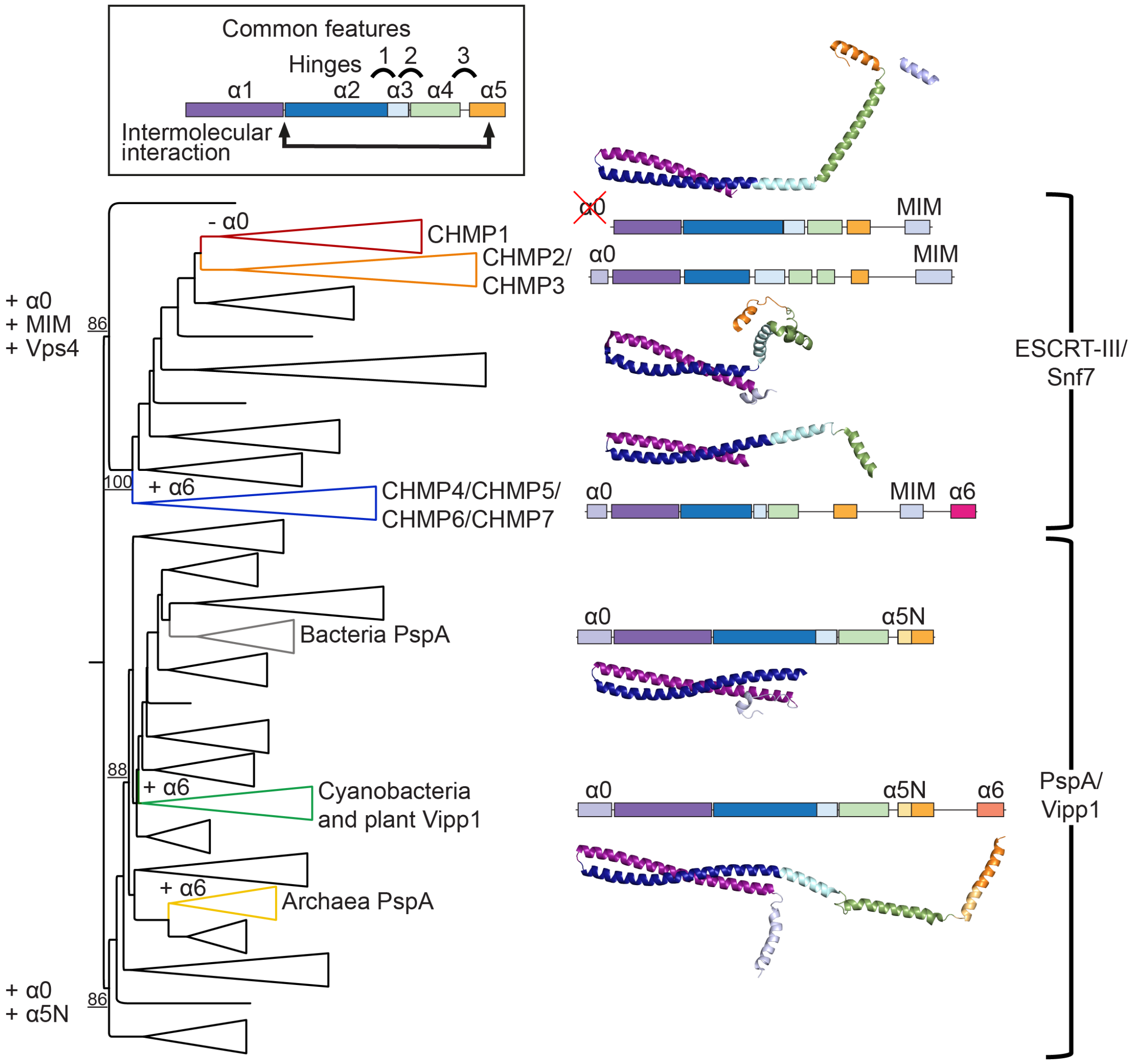

Data S1B. Phylogenetic tree presenting the relationship of the PspA/Vipp1 and ESCRT-III/Snf7 families. A few bootstrap values (numbers underlined) are shown. An equivalent tree with a more complete annotation is shown in Dendrogram S1. A scheme on the top shows the common characteristics (secondary structure elements, flexible hinges and conserved intermolecular interaction binding sites) present in all polypeptides in the resulting superfamily that includes all known PspA and ESCRT-III proteins and their homologues. Structural elements and protein sub-regions presumably acquired or lost by particular lineages during evolution are indicated by labels (α0, α5N, α6, MIM), along with Vps4. Experimentally solved structures of members of this superfamily are presented and their secondary structure elements are highlighted. Several of these structures were obtained from

protein fragments and do not include specific sub-regions. PDB accession codes from top to bottom: human CHMP1B (PDB 6TZ4); yeast Vps24/CHMP3 (PDB 6ZH3); yeast Snf7/CHMP4 (5FD7); *E. coli* PspA (4WHE) and *N. punctiforme* Vipp1 (6ZW4).

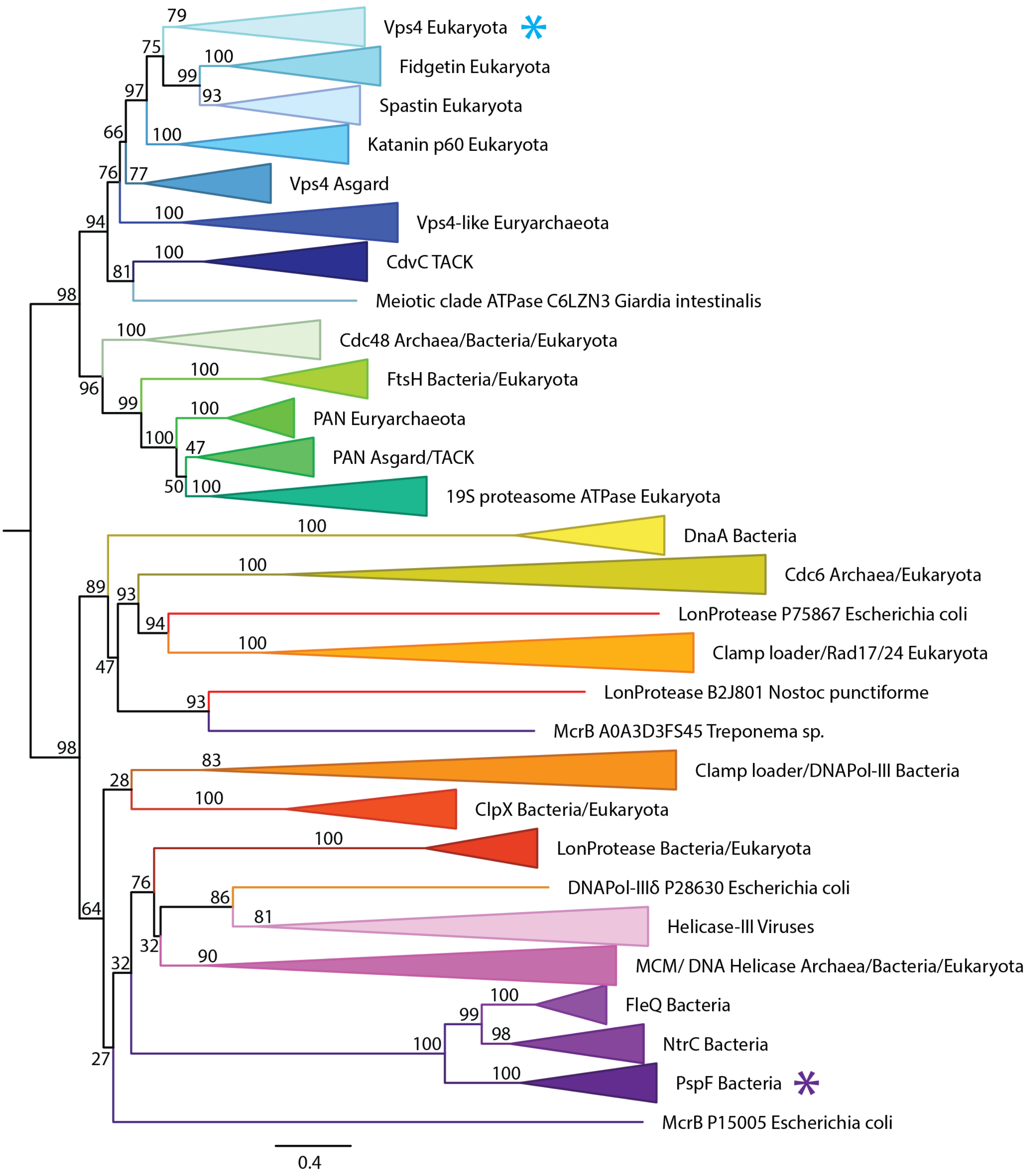

Data S1C. Phylogenetic tree presenting the evolution of AAA ATPases using 194 protein sequences. Numbers indicate the relative support from 100 bootstrap replicates. Scale bar represents expected substitutions per site. Note that eukaryotic Vps4 proteins (cyan asterisk) and their close homologues occupy a separate branch far from PspF (purple asterisk).
